# Well-designed protein amyloid nanofibrils composites as versatile and sustainable materials for aquatic environment remediation: A review

**DOI:** 10.1016/j.eehl.2023.09.003

**Published:** 2023-09-20

**Authors:** Xiaolin Zhang, Mamitiana Roger Razanajatovo, Xuedong Du, Shuo Wang, Li Feng, Shunli Wan, Ningyi Chen, Qingrui Zhang

**Affiliations:** a Hebei Key Laboratory of Heavy Metal Deep-Remediation in Water and Resource Reuse and Hebei Key Laboratory of Applied Chemistry, School of Environmental and Chemical Engineering, Yanshan University, Qinhuangdao 066004, China; b State Key Laboratory of Metastable Materials Science and Technology, Yanshan University, Qinhuangdao 066004, China; c College of Life & Environment Sciences, Huangshan University, Huangshan 245041, China; d College of Environment, Zhejiang University of Technology, Hangzhou 310014, China

**Keywords:** Amyloid nanofibrils, Composites, Preparation, Characteristics, Adsorption

## Abstract

Amyloid nanofibrils (ANFs) are supramolecular polymers originally classified as pathological markers in various human degenerative diseases. However, in recent years, ANFs have garnered greater interest and are regarded as nature-based sustainable biomaterials in environmental science, material engineering, and nanotechnology. On a laboratory scale, ANFs can be produced from food proteins via protein unfolding, misfolding, and hydrolysis. Furthermore, ANFs have specific structural characteristics such as a high aspect ratio, good rigidity, chemical stability, and a controllable sequence. These properties make them a promising functional material in water decontamination research. As a result, the fabrication and application of ANFs and their composites in water purification have recently gained considerable attention. Despite the large amount of literature in this field, there is a lack of systematic review to assess the gap in using ANFs and their composites to remove contaminants from water. This review discusses significant advancements in design techniques as well as the physicochemical properties of ANFs-based composites. We also emphasize the current progress in using ANFs-based composites to remove inorganic, organic, and biological contaminants. The interaction mechanisms between ANFs-based composites and contaminants are also highlighted. Finally, we illustrate the challenges and opportunities associated with the future preparation and application of ANFs-based composites. We anticipate that this review will shed new light on the future design and use of ANFs-based composites.

## Highlights

• The preparation strategies of amyloid nanofibrils (ANFs)-based composites are summarized.
• Links among preparation, morphologies, functional groups, and performance are studied.
• ANF-based composites are effective for the removal of inorganic, organic, and biological pollutants.
• The adsorption mechanisms between ANF-based composites and contaminants are highlighted.
• The challenges and opportunities of ANF-based composites are proposed.

## Graphical abstract

**Figure:**
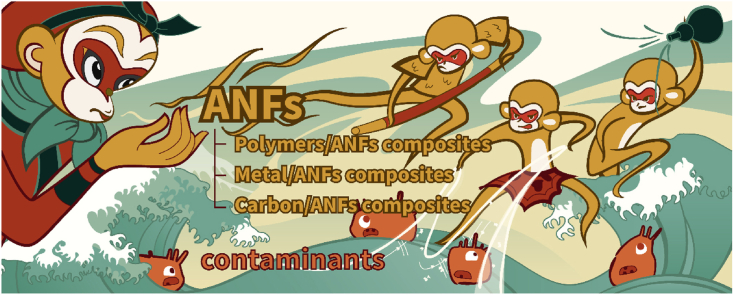

## 1 Introduction

Aquatic ecosystem pollution has become a major concern in recent years since various dangerous pollutants, such as heavy metals, organic compounds, and microbes from industries, agriculture, hospitals, and landfills are dumped into water environments at concentrations unfit for human consumption [1,2]. These pollutants harm drinking water quality and supply, putting public health at risk. Heavy metals, for example, can deplete the body’s essential nutrients, weakening the immune system and causing malnutrition-related diseases and growth retardation [3]. Organic pollutants are linked to skin lesions, embryotoxicity, teratogenicity, nephrotoxicity, hepatotoxicity, and cancer [4]. Transmission of pathogenic microorganisms through water can cause gastroenteritis [5]. Furthermore, between 2000 and 2050, population growth poses a new challenge to safe and sustainable water supply, and the world may face severe water scarcity [6]. Therefore, various water purification technologies have been proposed to increase the safe water supply.

Chemical precipitation, ion exchange, membrane separation, adsorption, and advanced oxidation/reduction processes (AOPs/ARPs) have all been widely used in wastewater purification technology to ensure safe and adequate drinking water [[7], [8], [9]]. While these methods are effective at removing pollutants, most of them are deemed unsustainable. For example, ion exchange has a relatively high operating cost [10]; membrane separation has a trade-off between selectivity and permeability [11]; and AOPs/ARPs have a pH limit and require large amounts of toxic catalysts [12,13]. Therefore, adsorption is worthy of consideration among the various water purification technologies available due to its design flexibility, low investment and operation costs, and minimal energy input [14]. Furthermore, various adsorbents have been utilized in multiple water technologies, such as bio-based adsorbents derived from byproducts or waste, resulting in almost no environmental footprint and significantly elevating their position in the value chain [15].

In recent years, amyloid nanofibrils (ANFs) have piqued the interest of scientists due to their environmentally friendly nature and natural availability. ANFs are supramolecular polymers with abundant β-sheet secondary structures featuring unique surface functionality and morphologies [16,17]. ANFs are widely used in tissue engineering, biomedicine, materials science, nanotechnology, and environmental protection because of their remarkable nanomechanical properties, polarities, and charges [18,19]. Furthermore, ANFs have clear fiber morphology, a high aspect ratio, rich functional groups, superb biodegradability, antibacterial properties, and high strength and stability [20]. The abundant functional groups can chelate with heavy metals and remove pollutants through electrostatic interactions [21]; high aspect ratio can provide more adsorption sites to efficiently remove organic and inorganic pollutants [22]; the antibacterial properties can play a role in bacterial anti-fouling [23]; high strength and stability make it possible to modify ANFs, which can further improve the removal performance of pollutants [24]. Therefore, ANFs can be applied to aquatic environmental remediation.

Various conventional protocols, such as one-step guanidine hydrochloride denaturation and heating denaturation, have produced ANFs [25,26]. Each protocol technique could substantially affect the physicochemical properties of ANFs, affecting the number of active sites and mass transfer processes. Pure ANFs have a limited adsorption capacity, and their colloidal nature causes separation and permeability issues. For this reason, studies have suggested the synthesis of ANFs with various functional materials, such as polymers and metals, to fulfill their applications [24]. Combining ANFs with other substances and support materials is a strategy for altering the chemical makeup of ANFs and improving their adsorption performance and application in water purification. However, the conventional synthesis technique for preparing ANFs-based composites has not yet been established.

Previous reviews have provided fundamental information on using ANFs-based composites for water purification. Peydayesh and Mezzenga, for example, emphasized the long-term viability of ANFs used for water purification on a large scale [27]. They also clarified the production of ANFs from diverse protein sources, such as food waste and byproducts of the cattle, dairy, and agriculture industries [28]. Gandavadi et al. summarized and discussed the removal of dyes, heavy metal ions, and other pollutants by bio-based nanofibers, which included a minor portion of protein-based nanofibers [29]. Yang et al. also reported the effectiveness of protein-based membranes in removing transition metal ions from wastewater due to the abundance of amino acid residues on the protein surface [22].

Research on the development of ANFs-based composites increases annually. However, to our knowledge, a systematic review of the application of various ANFs-based composites in water purification is lacking. This paper reviews the recent progress in removing contaminants from aquatic environments using ANFs and their composites. This review consists of a brief summary of the preparation technique for ANFs and their composites and a detailed discussion on the physicochemical properties (morphologies and functional groups) of ANFs-based composites to determine the regularities. The application for the removal of inorganic contaminants (heavy metals, noble metals, and radioactive substances), organic contaminants (dyes, antibiotics, and microplastics), and biological contaminants (bacteria and genes) are then highlighted. Moreover, we illustrate the adsorption mechanisms and conclude the review by highlighting the limitations of current research and providing an outlook for developing ANFs-based composites.

## 2 Preparation and characteristics of amyloid nanofibrils-based composites

Fabrication protocols and conditions substantially affect the physicochemical properties (i.e., morphologies and functional groups) of ANFs and ANF-based composites, which may in turn affect the ANF material adsorption performance. The subsequent section summarizes, analyzes, and discusses the synthesis and properties of pure ANFs and their composites, including polymers/ANFs, metal/ANFs, and carbon/ANFs.

### 2.1 Pure amyloid nanofibrils

Most proteins (>98.7%) contain at least one suitable self-complementary mini-sequence to form ANFs. In other words, a common feature of proteins is their ability to self-assemble into ANFs [30]. Protein feedstock for ANFs has microbial proteins and food source proteins [31]. Microbial-derived ANFs are usually expressed by bacteria, and in order to obtain superior heavy metal removal properties, the strains often need to go through the process of designing primers, polymerase chain reaction (PCR) amplification, purification of the gene fragments, and genetic recombination [32]. The cost of culturing an average colony is roughly 1.65 dollars/L, including petri dishes, peptone, sodium chloride, etc. Moreover, the engineered strain could be cloned to a large size, and the upfront cost can be ignored. Studies show that non-toxic food proteins are classified as animal, plant, and fungus proteins [33]. Animal proteins outperform plant proteins in terms of functionality and have higher yields, making animal proteins ideal raw materials, while proteins from plants and fungi have received little attention [34]. The upfront cost of animal-derived ANFs comes mainly from the cost of feeding animals such as cows and chickens, which is about 1,170, or 2.7 dollars. However, they can consistently produce raw materials for ANFs, causing a small increase in costs. Furthermore, the cost of food source ANFs is approximately 133 dollars/L, including protein powder, dialysis bags, etc. Although the raw material cost of microbial ANFs is lower, their preparation steps are more complicated than those of food-derived ANFs. Therefore, this review focuses on food-derived ANFs. ANFs can be formed from approximately 23 different food proteins; therein, ANFs from whey β-lactoglobulin (WβL), hen egg-white lysozyme (HEWL), and bovine serum albumin (BSA) proteins are commonly used in wastewater treatment (Table S1). Therefore, this section will focus on pure ANFs derived from WβL, HEWL, and BSA preparation protocols.

**Fig. 1:**
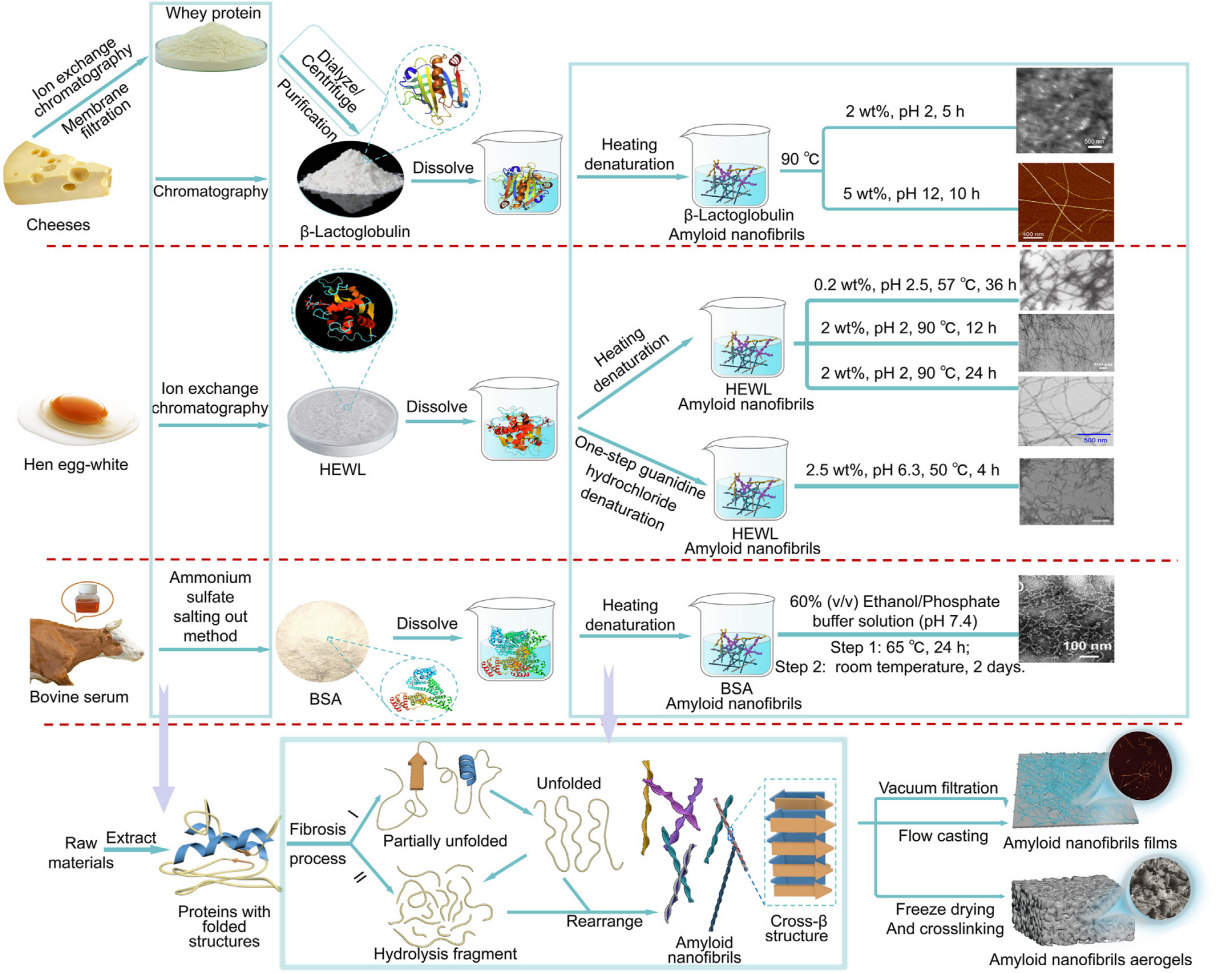
Schematic diagram of the preparation of amyloid nanofibrils [26,35,44,[52], [53], [54], [55]].

Purification and denaturation are the two foremost steps in ANFs’ preparation (Fig. 1). In the purification processes, various techniques such as chromatography, membrane filtration, salting out, electro-acidification, and combination were used [35]. Furthermore, dialysis is an essential purification step that should not be overlooked. Dialysis is a kind of membrane separation that removes small molecules (e.g., inorganic salts and monosaccharides) from the protein solution [36]. It requires a clean and low-temperature environment. Generally, the purification of 20 g of protein powder requires dialysis for 5–8 days, which takes a long time. In addition, ultrapure water in the external environment needs to be replaced regularly to remove small molecules and maintain the rotation of the external solution to speed up the dialysis process, which leads to a complicated preparation process [37]. Moreover, the yield of purified protein powder is about 30%–70%, requiring more productive purification methods.

Denaturation is the second step in the production of ANFs. This process is dominated by protein unfolding, misfolding, and aggregation via exposed hydrophobic sites, which are then converted into β-sheet-rich structures [38]. Protein conversion into AFNs depends on extrinsic conditions like temperature, protein concentration, solution pH, and heating time. Regarding temperature conditions, proteins are denatured at low temperatures and can be damaged at extremely high temperatures [39]. Therefore, the optimum temperature to prepare ANFs is 50–90 °C (Table 1). The protein concentration can also influence the β-sheet contents and ANFs’ morphologies. Studies have recommended using about 0.2 wt% to 5 wt% during fibrillization. For the pH condition, previous studies have shown that the operating pH must be far from the protein isoelectric point to promote peptide bond cleavage and electrostatic interactions [40]. In addition, pH adjustment is an effective method for controlling the morphology of ANFs. For example, as the pH increased from 2.0 to 5.8 to 7.0, the morphologies of WβL changed from rod-like structures to worm-like clusters [41]. Additionally, the heating time appears to affect the morphological characteristics of ANFs. For example, the WβL exhibited spherical aggregates with a 20–50 nm fibrous morphology and a length of up to 200 nm after 1 and 5 h of heating. In particular, after 10 h of heating, the ANFs exhibited an extremely high aspect ratio (∼10^3^) [42]. Most ANFs can reach lengths of several microns and have a smaller diameter than other fibers (carbon, α-Fe_2_O_3_, chitosan, etc.), giving them the distinct advantage of a high aspect ratio (Fig. S1).

**Table 1:** Fibrillization conditions and morphologies of amyloid fibrils.

Protein	Temperature (°C)	Concentrations (wt%)	pH	Time(h)	Diameter (nm)	Length (μm)	Ref.
Whey β-lactoglobulin (WβL)	90	5	12	10	7	Several	[42]
	90	2	2	5	5	3–4	[45]
	90	4	2	5	2–4	1–10	[41]
Hen egg-white lysozyme (HEWL)	57	0.2	2.5	36	15–30	Several	[46]
	65	0.2	2.7	72	10–20	Several	[47]
	50	1	6.3	4	10–20	–	[48]
	90	2	2	12	20	Several	[37]
	90	2	2	24	3–4	Several	[49]
	50	2.5	6.3	4	20	0.5–1.6	[25]
Bovine serum albumin (BSA)	65	1	7.4	24	10	–	[43]
	65	150 (μM)	7.4	6	22	1	[50]
	65	1	7.4	24	50	1	[51]

Various techniques can be used to transform ANFs into materials, such as biofilms, aerogels, hydrogels, etc. For example, ANFs can be assembled into uniform, transparent membranes using vacuum filtration or flow casting. The properties of ANFs are affected by their transformation into different forms. Wu et al. developed biofilms with BSA nanofibrils. They produced fibrils with a mean diameter of up to 10 nm, porous microstructures with pore sizes ranging from 5 to 150 nm, and a thickness of 95 μm [43]. Freeze-drying and cross-linking ANFs can generate aerogels. The WβL ANFs-based aerogels were extremely light [44].

**Fig. 2:**
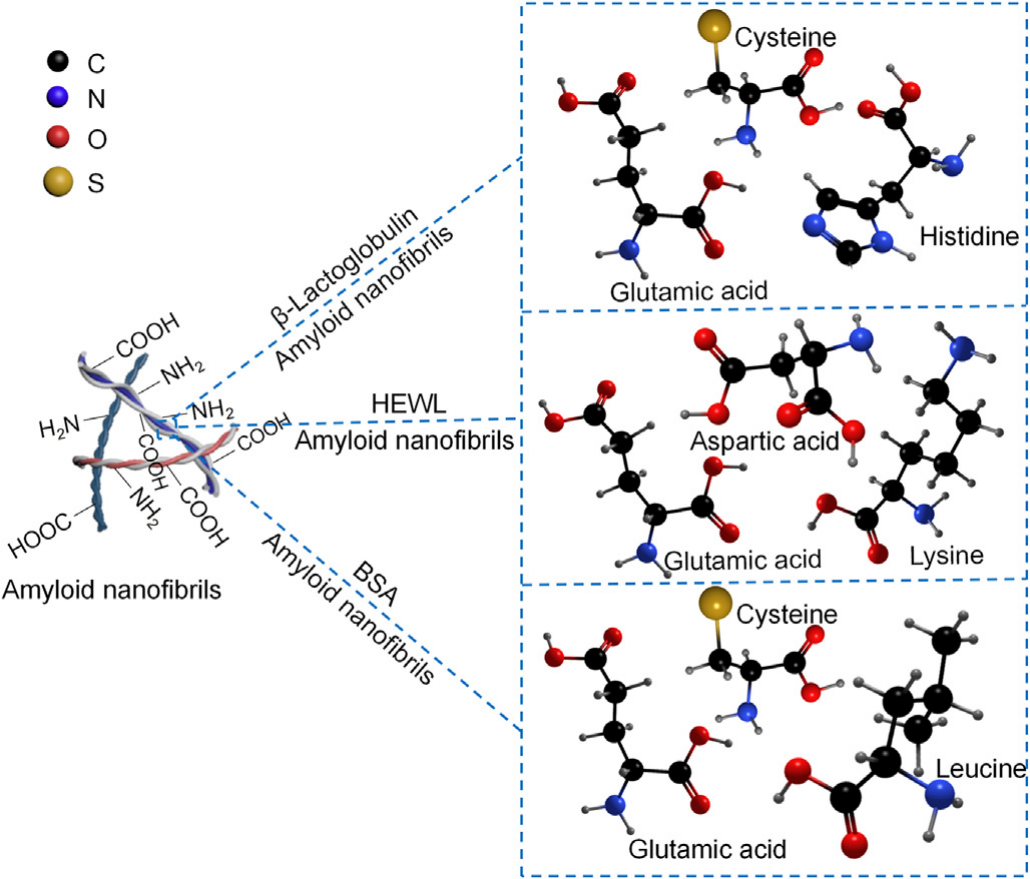
Main amino acids of typical amyloid nanofibrils [57,[62], [63], [64]].

The ANFs inherit the various amino acids found in proteins. Each amino acid responds differently to heavy metals. For instance, cysteine contains sulfhydryl groups, which can act as a natural reducing agent for heavy metal ions. Furthermore, cysteine residues are the primary amino acids capable of binding arsenic (As), mercury (Hg), and iron (Fe) [56]. Aspartic acid and glutamic acid are negatively charged amino acid residues that can attract positively charged lead (Pb) and nickel (Ni). Histidine residue also has a high binding affinity for Ni [57]. Positively charged lysine residues can attract negatively charged heavy metals. In addition, leucine is one of the most abundant amino acids in ANFs, contributing numerous amino and carboxyl functional groups. The WβL mainly contains cysteine, histidine, and glutamic acid; the HEWL possesses aspartic acid, glutamic acid, and lysine; and the BSA majorly carries cysteine, leucine, and glutamic acid (Fig. 2). The amino acid groups of ANFs comprise of –NH_2_, –COOH, and –R side chains. These amino acids are mainly characterized using X-ray photoelectron spectroscopy (XPS) and Fourier transform infrared spectroscopy (FTIR). The –NH_2_ functional group is the most prevalent amino group on WβL nanofibrils. In the XPS spectra of N 1s, peaks at about 399.8 eV and 399.2 eV corresponded to the imine and amine groups, respectively [58]. The ANFs derived from HEWL contain groups that could serve as active sites for metal ion binding, specifically –O–C=O, –N–C=O, –C–S, –C–O, –C–N, and –C–H [59,60]. BSA nanofibrils had amide I and amide II bonds, indicating the presence of hydrogen bonding. Furthermore, –S–S– and –SH groups were also found in BSA molecules, demonstrating disulfide bonding to form biofilms [61].

### 2.2 Polymers/amyloid nanofibrils composites

Polymers comprise an enormous number of identical and simple structural units joined together by high-molecular-weight covalent bonds [65]. Various polymers have been used to modify and improve the stability of ANFs. Polydopamine (PDA) and polyethyleneimine (PEI) alter the properties of ANFs. Furthermore, cellulose and chitosan are used as carriers to improve the ANFs’ stability. Table S2 summarizes the components, synthesis methods, morphologies, and functional groups of polymers/ANFs composites.

The mussel-inspired PDA has excellent adhesion properties and can be coated with various materials [66]. Liang et al. added solid dopamine hydrochloride to a lysozyme protofilament dispersion solution to induce, cross-link, and aggregate larger diameter fibrils, which are then vacuum filtered to produce PDA/ANFs membrane (Fig. S2a) [37]. They demonstrated that the diameter of ANFs increased from 20 nm to 80 nm after PDA modification due to the cohesion produced by PDA. The formation of porous three-dimensional structures resulted from the crossed stacking of nanofibers (Fig. S2b). The BET-specific surface area and the membrane thickness were 220.4 m^2^/g and 70.5 μm, respectively.

Liu et al. prepared lysozyme nanofibrils coated with PEI and bound with PDA to increase ANFs active sites (Fig. S2c) [25], since PEI was difficult to chemically graft to fibrils due to its water solubility and macromolecular structure [67]. The amyloid lysozyme fibrils (AFL)-PEI composite’s scanning electron microscope (SEM) image (Fig. S2d) showed three-dimensional hierarchical structures owing to the PEI conjugation. The network’s pore size ranged from 20 to 500 nm, while the diameters were 40–60 nm. Furthermore, PEI conjugations could generate greater primary amine (–NH_2_) functional groups, which increase AFL-PEI composite active sites. A previous study has also investigated the *in-situ* self-assembly of PEI/g-C_3_N_4_ and ANFs during lysozyme denaturation [47]. Adding g-C_3_N_4_ to PEI/ANF composites could strengthen their reduction performance. The PEI/g-C_3_N_4_@ANFs with the porous three-dimensional and chain-like network possessed an average particle size of 41.3 nm, an *S*_BET_ of 39.6 cm^2^/g, and a pore volume of 0.25 cm^3^/g. Besides that, Li et al. tuned the poly(N-isopropyl acrylamide) (PNiPAM) layers to control the viscoelasticity of ANF hydrogels while ensuring that the fiber structure was not damaged [68]. ANFs-PNiPAM had hydrodynamic radii and 640 nm and 22 nm thicknesses, respectively.

Cellulose and chitosan have also been implemented as ANF supports due to their large surface area, high mechanical strength, renewability, and low cost [69,70]. For example, cellulose and lysozyme nanofibril suspensions were mixed and stirred at varying dry mass ratios, and then the mixture was filtered and dried to produce bio-sorbent films [71]. The presence of cellulose on the membrane increased membrane flux. ANFs were uniformly dispersed in CNFs on the surface of films, increasing the mechanical stability of ANFs and cellulose composites over pure ANFs. At the same time, ANFs promoted cellulose bonding and increased adsorption sites. The porous cellulose-PDA/ANFs composite aerogel also exhibited exceptional mechanical strength due to the intermolecular and internalization bonding of the cellulose/ANFs network [72]. The membrane of cellulose/ANFs exhibited good resistance in strong alkali or acidic environments, which was critical in practical application [73].

The development of ANF membranes was hindered by biofouling. Increasing the membrane’s hydrophilicity with polymers could prevent biofouling [74]. Palika et al. have been using slow pressing and drying to produce hybrid membranes with varying proportions of chitosan, ANFs, and carbon [23]. The hybrid membrane’s active layer was thicker, denser, and significantly more hydrophilic than the ANF's pristine membrane.

Polymers can act as both modifiers and supports. Vacuum filtration or slow pressing can generate polymer/ANF composite membranes and gels. There are two types of morphology for polymer/ANFs composites: porous three-dimensional structure and thin flake shape. Due to the presence of ANFs and the addition of polymers, the polymers/ANFs composites contain a wide range of functional groups. For example, PDA introduces –N–H groups, whereas PEI conjugations generate more –NH_2_. Compared to pure ANFs, polymer-modified ANFs composites have pore sizes ranging from 20 to 500 nm and diameters between 10 and 80 nm. The polymer can also change the hydrophilicity and permeability of polymer/ANFs membranes and enhance their stability. Composite polymer/ANF research is still in its infancy. In the future, polymers such as polyaniline, polypyrrole, and polythiophene could be utilized in research [75].

### 2.3 Metal/amyloid nanofibrils composites

Metal nanoparticles are important functional materials in water treatment because of their high adsorption capacity and efficient catalytic activity due to the nano-size effect [76,77]. However, metal nanoparticles tend to aggregate and become inactive [78,79]. ANFs can use their abundant functional groups to stabilize metal nanoparticles [51]. The ultra-high aspect ratio of ANFs has the ability to generate tiny metal nanoparticles and increase metal loading capacity [80].

**Fig. 3:**
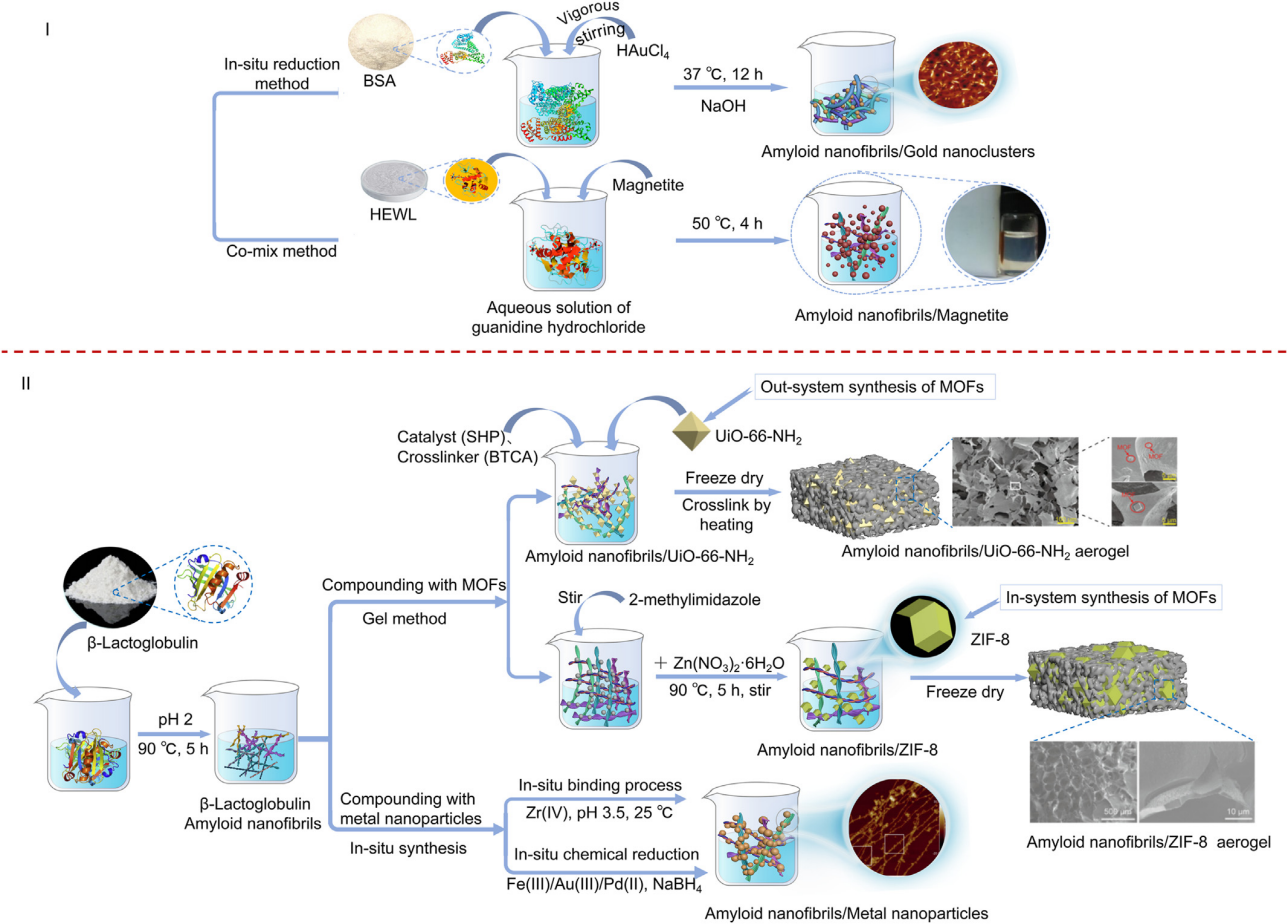
Schematic diagram of the preparation of metal/amyloid nanofibrils composites [24,45,81,88,89].

As shown in Table S2, the principal metals currently loaded by ANFs are ferroferric oxide (Fe_3_O_4_), zero-valent iron (Fe^0^), zirconium oxide (ZrO_2_), gold (Au), and palladium (Pd). The preparation of metal/ANFs composites mainly has two strategies, i.e., combination during the denaturation of protein and modification after the fibrillation (Fig. 3). For example, Leung et al. combined ANFs with Fe_3_O_4_ nanoparticles to improve recyclability by applying an external magnetic field [48]. The assembly conditions included mixing Fe_3_O_4_, hen lysozyme solution, and guanidine hydrochloride, adjusting the pH to 6.3 with potassium phosphate buffer, and stirring for 4 h at 50 °C. Au nanoclusters could be embedded in BSA nanofibers during alkaline denaturation [81]. Notably, BSA served as a stabilizer and a reducing agent. The –SH group of cysteine and a phenolic group of tyrosine reduced Au^3+^ to Au^+^. Similarly, ANFs could also reduce transition metals such as Cu^2+^ [82]. In most cases, ANFs were first produced and then metal-modified. Many amino acids (tyrosine, cysteine, and tryptophan) in ANFs could act as reducing agents to convert Fe^3+^ to Fe^2+^ [83]. Fe ions are strongly boundto ANFs by supramolecular coordination bonds [84]. Adding chemical agents for further processing was required to obtain the desired metal type. For example, the strong reducing agent NaBH_4_ has been used to ensure the formation of zero-valent metal nanoparticles [85]. Besides, alkali precipitation was a common way to obtain metal oxides. Zhang et al. anchored Zr^4+^ with the –NH_2_ group of ANFs. Afterward, a NaOH solution was added to form ZrO_2_ [86]. ANFs also form complexes with metal-organic frameworks (MOFs) such as UiO-66–NH_2_ and ZIF-8. MOFs had the characteristics of high surface area, tunable pore structure, and multiple active sites [87]. MOFs/ANFs composites mainly existed in the form of aerogel. Depending on the MOF type, they could be self-cross-linked or assisted by a cross-linking agent [88,89]. The fundamental principle was that neither should be undermined.

**Fig. 4:**
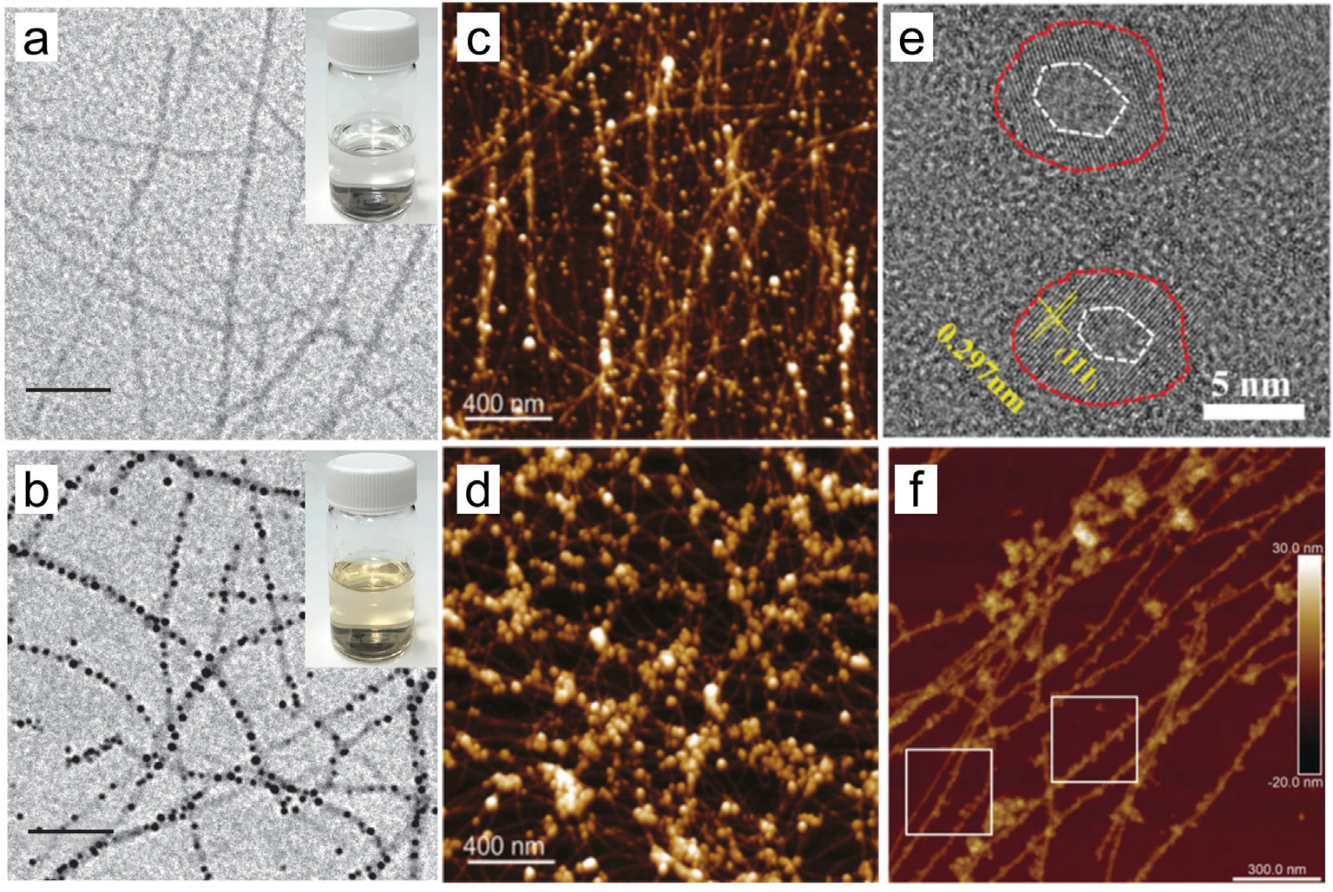
Transmission electron microscope (TEM) images of pure β-lactoglobulin (BLG) fibrils (a) and iron–BLG fibril nanocomposites (b), with insets showing their respective suspensions. Scale bars, 100 nm. Reproduced with permission from Ref. [83]. Copyright (2017) Springer Nature. 3D AFM image of β-lactoglobulin amyloid fibrils decorated with gold (c) and palladium (d) after the respective metal salt reduction by NaBH_4_. Reproduced with permission from Ref. [45]. Copyright (2015) American Chemical Society. (e) High-resolution TEM image of amyloid fibrils coated by ZrO_2_. (f) Atomic force microscopy (AFM) image of amyloid fibrils coated by ZrO_2_. Reproduced with permission from Ref. [86]. Copyright (2019) Wiley.

The morphologies of metal/ANFs composites are kept long and linear. The core feature was the uniform distribution of nanoparticles along the nanofibers. As shown in Fig. 4a and b, Fe^0^ nanoparticles with a 5–20 nm diameter were decorated on the surface of ANFs [83]. A recent study has also demonstrated that ANFs could prevent the rapid precipitation of nano-Fe^0^ into black clusters. Besides Fe nanoparticles, studies have also demonstrated that Au^0^ and Pd^0^ nanoparticles were tightly covered on the ANFs’ surface with a high linear grafting density (Fig. 4c and d) [45]; and a slight β-lactoglobulin fibril aggregation was induced by more Pd^2+^, forming clusters of large Pd nanoparticles. Yu et al. found that the graphene oxide embedded bovine serum albumin (GO-AuNCs@BSA) hybrid membrane had vast wrinkles, and lots of AuNCs were distributed on the surface of GO with a size smaller than 5 nm [81]. Similarly, Zhang et al. successfully prepared ZrO_2_/ANFs with a 13–18 nm diameter. Their high-resolution transmission electron microscope (TEM) results confirmed that the size of the embedded ZrO_2_ was 7 nm. Furthermore, in the crystalline phase, the distance between fringes was 0.297, which can be attributed to the interplanar spacing corresponding to the (111) plane of monoclinic ZrO_2_ (Fig. 4e) [86]. The dimensions of the fibrils were approximately 3–4 mm in length (Fig. 4f). The functional groups of metal/ANFs composites are primarily derived from ANFs, including –NH_2_, –COOH, –C=O, –N–H. Among them, the –NH_2_ group was the critical factor in grafting metal. Many simple metallic substances or compounds are also worth loading onto ANFs such as Cu^0^, Co_3_O_4_, TiO_2_, and so on [90].

### 2.4 Carbon/amyloid nanofibrils composites

Up to now, activated carbon (AC) has been the major carbon material combined with ANFs due to its high surface area, abundant pore space, and low production cost [91,92]. To overcome the bottleneck of difficult separation of ANFs suspensions, ANFs are adhered to AC to prepare a hybrid membrane, which gives full play to the viscosity of ANFs and the rigidity of AC [93,94]. Previous research has also prepared carbon/ANFs composites in aerogel form. The preparation methods, morphologies, and functional groups of carbon/ANFs composites are shown in Table S2.

Two simple steps were required to prepare a hybrid carbon/ANFs membrane. First, various ratios of ANFs and AC solutions were combined. Then, 0.22 μm cellulose filters were used for vacuum filtration to obtain films. For example, Bolisetty et al. mixed 2 wt% β-lactoglobulin fibril (0.5 mL) and 10 wt% AC dispersion (5 mL) and then filtered the mixture (1 mL) to obtain the black ANFs (2 wt%) composite membrane (Fig. S3a) [21]. In the higher-magnification image of SEM (Fig. S3b), ANFs on the surface of AC were resolved, showing that the uniform hybrid membrane was successfully formed. The length of fibrils in the hybrid film was 1 μm, and the diameter was about 4–6 nm [95]. Furthermore, the hybrid membrane had better mechanical properties than the pure ANFs membrane, leading to accelerated water penetration and resistance to current impact. Peydayesh et al. prepared carbon/ANFs aerogel via hydrothermal carbonization at 180 °C for 5 h [96]. The ANFs-based aerogel had a uniform pore and a highly stable framework. At the same time, the samples based on monomers were more fragile and collapsed easily (Fig. S3c). During the carbonization, the composite aerogel maintained a fibrillar structure, which presented a short and rough shape (Fig. S3d). To reduce the cost, using whey protein without purification instead of β-lactoglobulin was a simple and scalable way [97]. Adding cellulose pulp was more beneficial for practical applications. Fig. S3e shows the AC component and the cellulose scaffold. Although it is crucial to select cheap materials, carbon nanotubes, graphene, and carbon quantum dots cannot be ignored [98]. ANFs and their composites possess unique physicochemical properties, making them desirable candidates for removing pollutants from water.

## 3 Adsorption performance of amyloid nanofibrils based composites

### 3.1 Adsorptive removal of inorganic pollutants

#### 3.1.1 Heavy metals

Heavy metal ions harm animals, plants, and humans due to their low decomposition rate and high toxicity [99]. Various materials have been applied to remove heavy metals from aquatic environments [100]. Based on the available literature, it has been shown that ANFs and their composites can efficiently remove heavy metal ions such as Pb(II), Hg(II), and Cr(VI) from water. In order to highlight the performance of ANFs-based composites in removing heavy metals, we summarized the target heavy metal, initial concentration, pH, degradation efficiency, and adsorption capacity of ANFs composites in Table 2.

**Table 2:** Summary of inorganic pollutants removal by ANFs-based composites and other materials.

Adsorbent	Pollutant	Initial concentration (mg/L)	pH	Degradation efficiency (%)	Adsorption capacity (mg/g)	Ref.
β-lactoglobulin fibrils	Pb(II)	50	–	–	212	[42]
Lys-CNFs	Pb(II)	50	–	–	270.3	[37]
ANFs-AC	Pb(II)	65	3.7	99.97	–	[21]
AC	Pb(II)	30	2.0	83	24.9	[101]
GO	Pb(II)	25	7.0	76.3	19.07	[102]
Grafted dextrin	Pb(II)	–	6.0	–	80	[103]
Whey protein fibrils	Hg(II)	50	3.0	81	25	[104]
CNFs/LNFs	Hg(II)	0.05	11.0	99	–	[71]
GO-AuNCs@BSA	Hg(II)	120	–	90.45	–	[81]
Melanin-impregnated activated carbon	Hg(II)	5	5.0	84.59	–	[105]
PEI-g-C_3_N_4_ NSs@LFs	Cr(VI)	10	7.0	98	23.5	[47]
Ethylenediamine-modified ANFs	Cr(VI)	3	7.0	–	2.5	[63]
Melanin impregnated activated carbon	Cr(VI)	5	5.0	86.6	–	[105]
ANFs-AC	Pd(II)	1,000	–	–	112.5	[60]
BSA fibrils biofilms	Au(III)	1079	–	92.16	305.19	[43]
Pure cellulose membrane	Au(III)	5	–	–	25.9	[106]
ANFs-AC	Pt (IV)	0.135	–	99.98	–	[57]
ANFs-AC	Cs(I)	10	4.0	–	0.62	[107]
HMO	Cs(I)	1.3	4.0	–	0.11	[108]
ANFs-AC	Tc-99m	–	–	99.99	–	[97]
PTL-β-CD	U(VI)	1	6.0	–	9.03	[109]
ANFs-AC	As(V)	0.24	–	98.6	–	[95]
Chitosan	As(V)	2	4.5	–	30.8	[110]
ANFs-AC	Al(III)	81	7.0	98	10.4	[111]
CAF-Zr	F^−^	10	6.2	99.5	21.8	[86]

Lys-CNFs, lysozyme complex nanofibers; ANFs-AC, amyloid nanofibrils-activated carbon; AC, activated carbon; GO, graphene oxide; CNFs/LNFs, cellulose nanofibrils/lysozyme nanofibrils; GO-AuNCs@BSA, gold nanocluster, and graphene oxide embedded bovine serum albumin; PEI-g-C_3_N_4_ NSs@LFs, poly(ethyleneimine)-modified graphitic carbon nitride nanosheets and lysozyme fibrils; HMO, hydrous manganese oxide; CAF-Zr, amyloid fibril/ZrO_2_ nanoparticles.

Zhang et al. have investigated the adsorption performance of alkaline-activated ultrafine β-lactoglobulin (ABL) nanofibrils toward Pb(II). They found that ABL nanofibrils could adsorb 60 mg Pb/g within 1 min. Moreover, the maximum adsorption capacity reached 212 mg Pb/g [42], which was 2.65 times compared to grafted dextrin (80 mg Pb/g) [103]. AFL not only has a high affinity with Pb(II) but also has the potential to select Pb ions in the presence of Ca(II) coexisting ions, which is reflected by the high distribution value (∼3,200 mL/g), suggesting that ABL nanofibrils had outstanding selectivity (Fig. S4a). Besides the pure ANFs, the ANFs composite also exhibited outstanding performance toward Pb ions. For example, Liang et al. have used lysozyme-coated mussel-inspired polydopamine (PDA) nanofiber membranes (Lys-CNFs) with a surface area of 220.4 m^2^/g to sequester Pb(II). Their result showed that the maximum adsorption capacity of Lys-CNFs reached 270.3 mg/g, which was higher than their used control material, coconut shell-activated carbon (26.5 mg/g) [37]. The PDA in the Lys-CNFs membrane has a crucial role in lead sequestration since it can expand the diameter and improve the surface chemistry of lysozyme nanofibrils. Strong ionic strength can cause the PDA to shift from an extreme sloughing state to a loosely formed state, which facilitates the release of a considerable quantity of adsorption sites [112]. Additionally, Liu et al. prepared lysozyme-coated PEI (AFL-PEI) conjugated through PDA surface coating. Their results demonstrated that the AFL-PEI can rapidly, efficiently, and selectively sorb Pb(II). This outstanding performance was due to the density of –NH_2_ groups originating from PEI [25]. Accordingly, the dynamic adsorption of AFL-PEI indicated that AFL-PEI nanofibrils could be treated with 12,200 kg of Pb(II) wastewater per kg of adsorbent, and the Pb(II) exhausted adsorbent could be regenerated by using a binary 5% Ca(NO_3_)_2_ and 1% HNO_3_ solution as regenerant (Fig. S4b), suggesting its possible industrial applicability. Sorriaux et al. also used PDA-modified nanocellulose/amyloid (CpA) composite aerogel to remove Pb(II) [72]. CpA aerogel showed high adsorption capacity (91.7%) within 5 min, attributed to the porous structure and the combination of –NH_2_ groups and Pb(II). The adsorption efficiency reached 91.7% within 5 min. Moreover, at solution pH 7.0, –NH_2_ groups were deprotonated with a rising electronegativity, resulting in powerful electrostatic attraction to Pb(II). Compared to porous chitosan with 80.83% removal of Pb(II), the CpA aerogel adsorption efficiency increased by 10.87%, while the operating time decreased by 35 min, which proved the CpA aerogel had practical value in water treatment.

Besides Pb(II) removal, ANFs composites can also adsorb hypertoxic Hg(II). Silva et al. demonstrated that amyloid nanofibrils-activated carbon (CNFs/LNFs) biofilm could remove Hg(II) with an adsorption capacity up to 255.9 mg/g at pH 11.0 [71] due to the electrostatic attraction between ANFs and Hg(II). Moreover, CNFs/LNFs were used to adsorb Hg(II) from natural spring water. The removal efficiency reached 93.3%, and the effluent was lower than the limit value of Hg(II) in drinking water [1 μg/L of Hg(II), Fig. S4c] [113]. The authors proved that the adsorption mechanism was mainly chemical adsorption. However, when using melanin-coated polyvinylidene difluoride membrane to remove Hg(II), the adsorption capacity was only 9.2 mg/g [105], which was far lower than that of CNFs/LNFs biofilm.

The gold nanocluster and graphene oxide embedded bovine serum albumin (GO-AuNCs@BSA) hybrid membrane was also used to sequester Hg(II) ions [81]. GO-AuNCs@BSA had a high removal efficiency with an adsorption capacity of 3.28 × 10^−6^ mol. The removal was mainly due to the Hg^2+^-Au^+^ interactions, Hg^2+^-cysteine residue binding, and electrostatic attraction. In addition, the GO-AuNCs@BSA hybrid membrane could be reused seven times successively, showing the stable structure of GO-AuNCs@BSA.

The capability of ANFs composites to sorbed Cr(VI) has also attracted significant attention due to the high toxicity of Cr(VI) [114]. Rodríguez et al. used a hybrid membrane, which was produced with whey protein fibrils (WPF) and active carbon (AC), to remove Cr(VI) [104]. They found that the adsorption capacity of Cr(VI) was 11.2 mg/g at an initial concentration of 100 mg/L (Fig. S4d). The interaction mechanisms between AC-WPF hybrid membranes and Cr(VI) ions were mainly due to the electrostatic attraction with WPF and the physical adsorption of AC. Furthermore, PEI-modified graphitic carbon nitride nanosheets and lysozyme fibrils (PEI-g-C_3_N_4_ NSs@LFs) adsorbents have been used to remove Cr(VI), and the removal efficiency reached 98% at pH 7 [47], which was higher than melanin-impregnated activated carbon (86.6% at pH 5) [105]. The high adsorption capacity is due to the electrostatic attraction between the positive charge –NH_2_ groups in PEI-g-C_3_N_4_ NSs@LFs and the negative charge of HCrO_4_^−^ or CrO_4_^2−^. Moreover, the hydrogen bonding between the lysozyme fibrils (LFs) provided by –COOH groups in LFs and HCrO_4_^−^ and CrO_4_^2−^ has an essential role in adsorption. Additionally, the adsorption isotherm and kinetics data fitted well with Freundlich and pseudo-first-order, indicating that physical adsorption was dominant. Furthermore, the adsorption capacities of LFs, PEI-g-C_3_N_4_ NSs, and ethylenediamine-modified ANFs were 9.81 mg/g, 14.3 mg/g, and 2.5 mg/g, respectively, which were lower than those of PEI-g-C_3_N_4_ NSs@LFs. ANF composites could also efficiently adsorb copper, Ni, and other heavy metals [80,115].

#### 3.1.2 Noble metals

With the decrease or depletion of mineral resources, how to recover noble metals from industrial wastewater such as Au, Ag, Pt, and Pd, is one of the current research hotspots [116,117]. ANFs-based composites could be employed to recover precious metals due to their excellent selectivity and high adsorption capacity. They were also applied to remove toxic precious metals for wastewater purification. The adsorption mechanisms mainly included physicochemical actions, electrostatic interactions, and metal ion chelation or complexation. For instance, ANFs-AC hybrid membranes could adsorb Au(I) and Pd(II), reducing the concentration of Au(I) in industrial wastewater from 30 mg/L to 0.105 mg/L and the concentration of Pd(II) from 12.2 mg/L to 0.019 mg/L [21]. The recovery efficiencies reached 99.60% and 99.84%, respectively. The adsorption mechanisms were attributed to the interaction between amino acid residues of ANFs and noble metals and the physical process of AC. Furthermore, the adsorbed Au, Pd, and Ag were converted into valuable nanoparticles or films via high-temperature or chemical methods, which is an indispensable step in recycling. Similarly, Yang et al. used an amyloid-like protein membrane to remove multiple noble metal ions including Au, Pd, Pt, Ir, Os, and Ru [60]. As shown in Fig. S4e, the adsorption capacity towards Au was 1,034.4 mg/g, 3–15 times higher than that of AC, MOFs, ion exchange resins, etc. The mechanisms contained electrostatic interactions, metal ion complexation, and in situ reduction on the membrane surface. Furthermore, the 23 K Au (95.8 wt%) could be obtained by pyrolyzing the absorbed gold. Compared with a pure cellulose membrane (25.9 mg/g), the adsorption capacity of an amyloid-like protein membrane was increased by nearly 40 times, which showed the excellent adsorption performance [106].

#### 3.1.3 Radioactive substances

The nuclear power industry and hospitals produce wastewater with radioactive substances, destroying the human body’s central nervous, neuroendocrine, and blood systems and even causing death [118]. The ANFs-AC hybrid membrane was the primary ANFs-based composite applied to remove radioactive substances and had high adsorption efficiency (Table 2). Specifically, the radioactive pollutants included uranyl acetate, phosphorus-32, technetium (Tc-99m), iodine (I-123), iodine (I-131), gallium (Ga-68), lutetium (Lu-177), and cesium (Cs). The adsorption efficiencies for uranyl acetate and phosphorus-32 were 99.35% and 99.88%, respectively [21]. The adsorption sites and coordination effects could explain the removal mechanisms. Likewise, Tc-99m, I-123, Lu-177, and Ga-68 were also selected as targets, and the removal efficiencies towards them all exceeded 99.8% [97]. When treating hospital wastewater containing the mixture of Lu-177 and I-131, the efficiencies were up to 99.995% and 99.95%, respectively. The efficiencies of the cellulose-carbon membrane were only 34.5% and 3%, which were considerably lower than those of the ANFs-AC hybrid membrane. For the removal of Cs, the decontamination efficiency was up to 99.7% [107]. The mechanism was a supramolecular interaction between the cation Cs and ANFs based on β-lactoglobulin. Uranium (U) ions were removed by the composite of phase-transitioned lysozyme and β-cyclodextrin (PTL-β-CD) with an adsorption quantity of 1.405 mg/(g·m^2^) [109]. Compared to the competing cation vanadium, the adsorption ratio of U ions exceeded about 80 times (Fig. S4f). In the presence of conventional anions, the removal efficiencies decreased very little (Fig. S4g). PTL-β-CD was harmless to the environment owing to its enzymatic degradability and excellent biocompatibility.

#### 3.1.4 Other inorganic pollutants

As is a kind of metalloid. Most As environmental pollution comes from human activities. Millions of people will be poisoned if they consume water containing As [119]. The composite membrane of ANFs and AC (ANFs-AC) could remove both As(III) and As(V) with an efficiency of 98.6% [95]. After several reuses, the adsorption efficiency of this membrane did not decrease. The ANFs-AC was also prepared into different filters to adsorb As(III) and As(V) from Peruvian drinking water [94]. As shown in Fig. S4h, the result showed that the final concentration of As reached the World Health Organization standards, which benefited the 15 million target population worldwide. Higher aluminum (Al) intake might cause sterility in birds, make fish poisonous, affect the human nervous system, and so on [120]. The ANFs membrane could separate Al from the aqueous medium, and the efficiency was up to 98% [111]. The adsorption limits were 1,593, 637, and 2367 mM for AlCl_3_, Al(NO_3_)_3,_ and Al_2_(SO_4_)_3_, respectively. The adsorption capacity of the ANFs membrane was 10 times higher than that of the AC membrane (Fig. S4i). The electrostatic attraction and interaction between Al and amino acid residues of ANFs were dominant mechanisms. Excessive fluoride ion (F^−^) ingestion could lead to dental or bone fluorosis, seriously threatening human health [121]. Zhang et al. developed an amyloid fibril-carbon-nano ZrO_2_ hybrid membrane (CAF-Zr) to absorb F^−^ from wastewater and drinking water [86]. The efficiency was beyond 95%, and the ion distribution coefficient was up to 6,820 mL/g, 180 times higher than commercial ion exchange resins. Moreover, the adsorption capacity reached 21.8 mg/g, greater than the carbon matrix’s (1.88 mg/g). Furthermore, the CAF-Zr membrane possessed high selectivity for F^−^ under coexisting ions (CO_3_^2−^, Cl^−^, SO_4_^2−^, NO_3_^−^, Ca^2+^, and Na^+^). The mechanisms included the complexation between Zr and F^−^ and the classic hard-soft acid-base (HSAB) principle: Zr as a hard acid and F^−^ as a hard base.

### 3.2 Adsorptive removal of organic pollutants

The various organic pollutants produced in industries and agriculture, such as dyes, phenolic compounds, microplastics, pharmaceuticals, etc. have certain toxicity and carcinogenicity, seriously affecting the aquatic ecosystem and human health [122]. Multiple materials were developed for the adsorption of organic pollutants such as carbon, metallic oxides, silicon, and clays [123]. Currently, ANF-based composites have attracted widespread attention due to their greenness, high aspect ratio, and excellent adsorption performance. We summarized the removal conditions and results (initial concentration, pH, degradation efficiency, and adsorption capacity) of organic contaminants by ANFs-based composites and other similar materials, as listed in Table 3. The adsorption process was sometimes accompanied by flocculation, coagulation, or catalysis. The composites removed organic pollutants mainly in the form of membranes, fiber solutions, and aerogels. Furthermore, the mechanisms were primarily electrostatic attraction, hydrophobic interaction, hydrogen bonding, and π-π interactions.

**Table 3:** Summary of organic pollutants removal by ANFs-based composites and other materials.

Adsorbent	Pollutant	Initial concentration (mg/L)	pH	Degradation efficiency (%)	Adsorption capacity (mg/g)	Ref.
ANFs	Polystyrene	10,000	7.0	98.2	–	[26]
ANFs membrane	PFASs	0.4	7.0	96	–	[93]
PAC	PFASs	0.1	8.3	85%	–	[124]
ANFs aerogel	BPA	1	9.0	78	50.6	[44]
HEWL	RB-5	108	3.0	99	155	[48]
HEWL	AR-88	40	7.0	99	–	[46]
PLS	CR	200	6.0	100	2,000	[125]
Surface quaternized cellulose nanofibrils	CR	–	–	–	664	[126]
CpA	CV	10	–	93.1	18.7	[72]
LYS/TOCN	MeB	–	–	99.6	–	[73]
Cellulose nanocrystals	MeB	5	–	26	–	[106]
ANFs/ZIF-8	CV	10	–	94.2	202	[88]
Meldrum's acid modified cellulose nanofiber	CV	10	7.0	–	3.984	[127]
ANFs/carbon aerogel	RhB	–	–	–	125.2	[96]

PAC, powder activated carbon; HEWL, hen egg white lysozyme; PLS, porous lysozyme skeletons; CpA, the cellulose nanofibrils surface is coated with pDA and amyloid nanofibrils; LYS/TOCN, lysozyme/TEMPO-oxidized cellulose nanofibers; ZIF-8, zeolitic imidazolate framework-8; PFASs, polyfluoroalkyl substances; BPA, Bisphenol A; RB-5, Reactive Black 5; AR-88, Acid Red 88; CR, Congo Red; CV, Crystal Violet; MeB, Methylene Blue; RhB, Rhodamine B.

When using ANFs of HEWL to remove anionic dyes [Reactive Black 5 (RB-5) and Acid Blue 29] and cationic dyes (Victoria Blue B), the efficiencies were more than 90%, owing to electrostatic attractions and hydrophobic interactions [48]. The adsorption equilibrium could be reached within 15 min. Morshedi et al. chose acid red 88, direct violet 51, RB-5, and congo red as representative azo dyes [46]. The removal efficiencies of HEWL ANFs all exceeded 95%, far higher than those of AC (below 50%). Due to the presence of the charge, the electrostatic attraction between dyes and ANFs was influenced by pH, and the optimal pH was neutral. Likewise, ANFs based on β-lactoglobulin had outstanding removal efficiencies for ibuprofen (IBP, 98%), bentazone (92%), and bisphenol A (BPA, 78%), and the maximum adsorption capacities were 69.6, 54.2, and 50.6 mg/g, respectively [44]. Their adsorption kinetics were consistent with the pseudo-second order, indicating that chemical adsorption was dominant. Hydrophobic interactions, π-π stacking interactions, and electrostatic attractions between organic pollutants and the ANFs aerogel were possible mechanisms. In addition, the pH value affected the adsorption capacity because it changed the net charge of the aerogel and contaminants (Fig. S5a). The ANFs aerogel maintained excellent adsorption performance during three consecutive regeneration cycles, showing its remarkable reuse capability. Wu et al. observed that the removal efficiencies of methylene blue and 4-aminoantipyrine by BSA biofilms were beyond 90%, which is nearly 3.5 times higher than cellulose nanocrystals (26%) [106], and the adsorption capacity of rhodamine 6G reached 109.04 mg/g [43]. Furthermore, the biofilms could be recycled up to five times without obvious decay in their adsorption capacity (Fig. S5b), indicating that BSA biofilms with powerful capture ability could be applied to practical water treatment. The ANFs were indeed excellent adsorbents.

The CpA composite aerogel was prepared to remove trace organic compounds and dyes [72]. Ibuprofen (IBP), bisphenol A (BPA), and atrazine were the typical trace organic compounds with removal efficiencies of 76.7%, 65.8%, and 28.8%, respectively. The adsorption equilibrium was reached quickly within 15 min at an initial concentration of 1 mg/L, attributed to the rich adsorption sites and functional groups of the composite aerogel. Moreover, when extracting malachite green, crystal violet, rhodamine blue, acriflavine, acid fuchsin, and methyl orange, the efficiencies were all kept between 60% and 90% within 1 h (Fig. S5c). Compared with Meldrum’s acid-modified cellulose nanofiber, which is also a bio-based adsorbent (3.984 mg/g) [127], its adsorption capacity was increased by nearly 3.6 times (18.7 mg/g). The binding functions included electrostatic attraction, π-π interactions, and hydrogen bonding between dye molecules and the aerogel. These results showed that the CpA composite aerogel was a promising adsorbent for organic contaminants. ANF-based membranes were one of the most common ways to remove organic pollutants. When treating wastewater with PFASs by the ANFs membrane based on WβL, the findings indicated that the removal efficiencies of long-chain per- and polyfluoroalkyl substances (PFASs, >90%) were higher than short-chain ones (<80%) (Fig. S5d) [93], which was attributed to the stronger hydrophobicity of long-chain ones. Moreover, strong electrostatic forces could be provided at low pH (<5) conditions to promote the adsorption of PFASs. The researchers further explored the adsorption performance of ANFs-AC membranes for PFASs. The removal efficiencies of perfluorobutyric acid with low molecular weight were up to 96%, indicating the outstanding separation ability of the hybrid membrane. To facilitate the recycling of ANFs, magnetic nanoparticles were modified on the lysozyme [48]. The removal efficiencies of dyes by the decorated ANFs were still up to 92%–99% after operating 20 adsorption/desorption cycles, indicating the superior stability of magnetite-bound ANFs. Sometimes, the function of metal nanoparticles (Pd and Au)-decorated ANFs membranes was catalysis, thus reducing 4-nitrophenol into 4-aminophenol [45]. The flow-through mode based on ANFs composites achieved continuous catalysis and put forward a new train of thought for removing organic pollutants through synergistic adsorption and catalysis.

Microplastics (MPs) threaten the ecosystem and human health [128]. There is an easy way to remove MPs and humic acid (HA) by adding ANFs directly, according to mechanisms of coagulation and flocculation [26]. When using ANFs as flocculants, the removal efficiencies of polystyrene particles and HA were 93.4% and 61.9%, respectively. The reaction process showed that flocs began to form and precipitate after putting ANFs into MPs’ suspension. The electrostatic interaction played an essential role in this process, and the ANFs had maximum positive charge at pH 2, which increased the electrostatic interaction. However, for the removal of HA, the electrostatic interaction was the strongest in the neutral range. Compared to commercial flocculants (FeCl_3_·6H_2_O and polyaluminum chloride), the performance of ANFs was better (Fig. S5e). This method saved more materials that were difficult to biodegrade and environmentally unfriendly. Like many flocculants, ANFs could also remove suspended particles from aquatic environments, thus reducing turbidity and organic matter content.

### 3.3 Adsorptive removal of biological pollutants

Many countries worldwide still have problems with the biological contamination of drinking water [129]. Bacterial materials are biological pollutants, such as *Escherichia coli* (*E. coli*), *Salmonella*, *and Legionella*, etc., which cause bacterial diseases and threaten human health [130]. Bacteria removal mainly depends on the size exclusion mechanism, including microfiltration and nanofiltration, and disinfection like chlorination and ultraviolet irradiation [131]. However, pressure-driven membrane processes disrupted the cell membrane of pathogenic bacteria [132] and released genetic materials containing antibiotic-resistant genes (deoxyribonucleic acid, ribonucleic acid, and so on) [133]. Disinfection methods inactivate the pathogens but do not remove genetic material (antibiotic-resistant genes) [134]. Amyloid hybrid membranes are environmentally friendly, less costly, less energy-intensive, and can remove bacteria through size exclusion mechanisms. Moreover, they can adsorb genetic material due to hydrogen bonding, hydrophobic, van der Waals, and electrostatic interactions between the positively charged groups of ANFs and the negatively charged nucleic acids. In addition, it can improve membrane fouling resistance through the coating of polymers [23]. In order to show the advantages of ANFs-based membrane filtration more clearly, we compared membrane filtration based on ANFs and other methods of bacteria removal in Table S3.

Palika et al. systematically studied the removal efficiency, mechanism, and repeatability of bacteria and genetic substances by the filtration of membranes based on ANFs [23]. The results indicated that the removal efficiency of *E. coli* by the hybrid membrane (CCAHM) made of cellulose, AC, chitosan, and ANFs was up to 99.999%, which was attributed to the disruption of the bacterial outer cell membrane by –NH_2_ of chitosan and a size exclusion process. Furthermore, the removal efficiency of genetic material by CCAHM exceeded 99%, higher than that of the composite membrane composed of cellulose, AC, and ANFs. The composite of amyloid and layered double hydroxide (LDH) exhibited bactericidal activity for Gram-positive *S. epidermidis* [135]. The researchers found that the bactericidal activity is attributed to amyloid because the LDH could not get rid of bacteria. When amyloid was loaded onto LDH, its performance decreased due to the reduction of the number of exposed enzyme sites. However, the bacteriostatic properties remain. The above results demonstrated ANFs-based composites had great potential for efficiently removing bacteria and genetic materials from aquatic environments.

## 4 Adsorption mechanisms of amyloid nanofibrils based composites

**Fig. 5:**
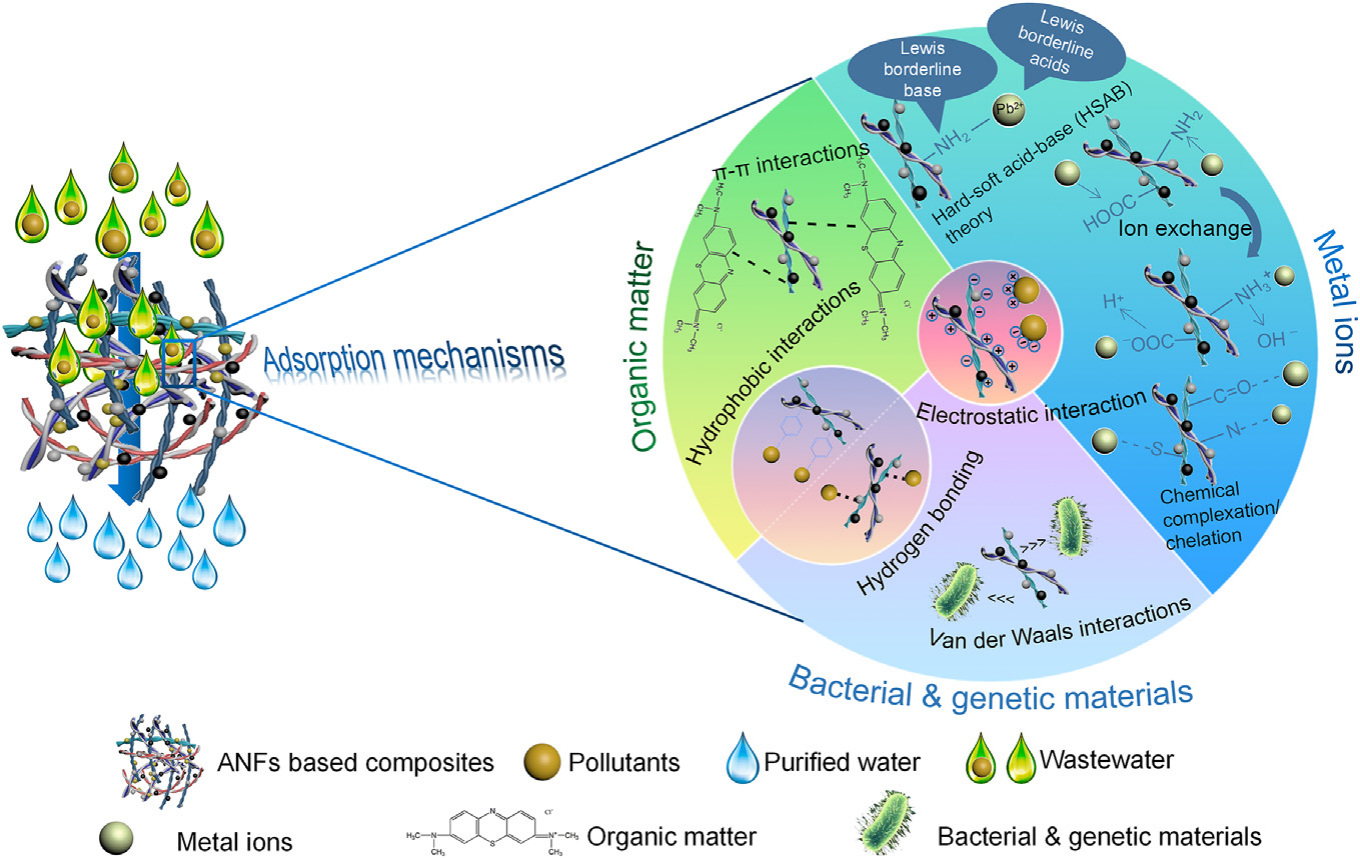
The possible adsorption mechanisms of pollutant removal by amyloid nanofibrils based composites.

ANF-based composites could adsorb water contaminants selectively and efficiently. Adsorption mechanisms are the key to controlling adsorption processes. Notably, ANFs-based composites’ adsorption mechanisms varied among various contaminants (Fig. 5). For metal ions, the main mechanisms included chemical complexation/chelation, electrostatic interaction, HSAB theory, and ion exchange. Organic matter interacted with ANFs-based composites through electrostatic interaction, hydrophobic interactions, hydrogen bonding, and π-π interactions. For bacterial and genetic materials, electrostatic interactions, hydrophobic interactions, hydrogen bonding, and van der Waals interactions dominated the mechanisms. Concretely speaking, chemical complexation/chelation benefited from the abundant –COOH and –NH_2_ groups of the ANFs-based composites, which were coordinated with metal ions to form N-metal and C=O-metal complexes [42]. The electrostatic interaction was attributed to the free ionizable –COOH and –NH_2_ groups, which could generate positive or negative charges to attract ionic pollutants, including organic matter such as dyes, humic acid, microplastics, negative DNA, and metal ions like Pb(II), Hg(II), and Cr(VI) [136,137]. Ion exchange was due to the exchange of H^+^ and OH^−^, which was generated from –COOH and –NH_2_ in the water environment, with a metal cation and metal anions. Hydrophobic interactions were attributed to the interaction between certain hydrophobic domains of the ANFs-based composites and the benzene ring of organic pollutants [89]. Furthermore, when the organic matter was in molecular form, intermolecular forces were generated between this pollutant and hybrid ANF-composites, which contained π-π interactions, hydrogen bonding, and van der Waals interactions [23,125]. The HSAB principle is also one of the theories to explain adsorption, which meant that hard acids and hard bases, as well as soft acids and soft bases could form stable compounds. For instance, the –NH_2_ (boundary base) from ANFs-based composites could combine with Pb(II) (boundary acid) to achieve the removal [61].

## 5 Conclusions and outlooks

Heavy metals, organic compounds, microbes, and other pollutants tremendously impact the aquatic environment and human health. In order to address this concern, researchers have developed bio-based materials such as amyloid nanofibrils (ANFs) to ensure safe water quality. ANFs are pathological hallmarks of many human diseases, typically formed by soluble protein misfolding and aggregation. ANFs and their composites have caught the interest of researchers in recent years due to their straightforward preparation technique, high aspect ratio, good chemical stability, biodegradability, and excellent adsorption capacity.

Purification and denaturation processes were widely used in the laboratory to generate ANFs. Temperature, pH, and protein concentration all play a significant role in the fibrillization of ANFs during the denaturation process. High temperatures (50–90 °C) and low pH can produce nanofibrils with diameters of 3–30 nm and lengths of several micrometers. ANFs possess a variety of amino acid groups on their surface, allowing them to synthesize with polymers, metals, and carbon. Combining ANFs with the previously stated materials could alter the physicochemical properties of ANFs and improve their removal performance. Depending on the synthesis method and intended application, the ANFs-based composites could be manufactured in various forms, including membranes, aerogels, and hydrogels.

Remarkably, when compared to pure ANFs, ANFs-based composites have advantageous properties, particularly increased binding sites, which could play an important role in the application of pollutant removal. For instance, polymer/ANFs composites exhibit a porous three-dimensional morphology with fibril diameters ranging from 10 to 50 nm. Furthermore, metal/ANFs composites exhibit fibrous structures decorated with spherical metal nanoparticles ranging in diameter from 5 to 20 nm. In addition, carbon/ANFs composites are fabricated into membranes with an average pore size of 2 μm.

ANFs-based materials have a high adsorption performance and can efficiently, rapidly, and selectively remove heavy metal ions, dyes, and radioactive substances from water. Carbon/ANFs composites have a high mechanical strength and can overcome membrane fouling. ANFs-based composites outperform traditional adsorbents regarding stability, mechanical strength, expandability, and reusability. Furthermore, the preparation of the ANF-based composites is both inexpensive and energy-intensive.

Various techniques, including XPS and FTIR, have investigated the interaction mechanism between ANFs and pollutants. The main interaction mechanisms between ANFs, metals, and organic pollutants are electrostatic interaction, chemical chelation, classic HSAB theory, hydrophobic interaction, and hydrogen bonding. The interaction mechanisms are attributed to the numerous functional groups (–NH_2_ and –COOH) derived from ANFs amino acid residues.

Despite the advantageous properties of ANFs and their composites, the following drawbacks are yet to be overcome.

(1) Most ANFs are derived from animals, which is easy to extract and has a higher yield than plant protein and bacterial protein, but at the same time increases raw material costs and manufacturing costs. Exploring more cost-effective raw materials for ANFs, including microbial-derived proteins and proteins from other sources, is also a key focus for us in the future.

(2) A lengthy purification process and a specific pH were required to prepare ANFs with a high aspect ratio. Therefore, it is necessary to develop innovative and suitable techniques. The novel technique should consider how to increase the rotation speed, how to reduce the dialysis time, and what inducer should be used to expedite the fibril formation. Furthermore, the morphology of ANFs, specifically the aspect ratio, should be retained when ANF composites are synthesized.

(3) Due to the low purification efficiency and low yield of protein powder that can induce nanofibril formation, a high-yield purification method must be explored.

(4) The good affinity of ANFs-based composites with heavy metal ions and organic contaminants allows us to investigate the performance of ANFs-based composites to remove heavy metal complexes (HMCs), thereby contributing to the development of relevant HMC removal technologies and the comprehension of the removal mechanisms and behavior of HMCs in environmental chemistry.

(5) To effectively separate ANFs and their composites from water, several studies have created ANFs composites in the form of a membrane, a magnetic fibril solution, and aerogel materials. However, only carbon-ANF membranes have been mass-produced for practical applications, with other forms still undergoing laboratory testing. As a result, industrial-scale production of other forms of ANFs composites, such as aerogel, will be required in the future. Future research should also consider the practical application of AFNs in large-scale water treatment using real wastewater.

## Author contributions

X.L.Z: conceptualization, investigation, writing–original draft, writing–original revise. M.R.R.: writing–original draft. X.D.D: conceptualization, investigation, writing–original draft, supervision, writing–review & editing. S.W., L.F., S.L.W., N.Y.C.: writing–review & editing. Q.R.Z.: conceptualization, supervision, project administration, writing–review & editing.

## Declaration of competing interests

The authors declare that they have no known competing financial interests or personal relationships that could have appeared to influence the work reported in this paper.
