# Supplementary material for: Well-designed protein amyloid nanofibrils composites as versatile and sustainable materials for aquatic environment remediation: A review
Source: Eco Environ Health. 2023 Sep 20;2(4):264–77. doi: 10.1016/j.eehl.2023.09.003 (PMC10902511; doi:10.1016/j.eehl.2023.09.003)
Supplement: Multimedia component 1 [file mmc1.docx]

**Supporting Information**

**Well-designed protein amyloid nanofibrils composites as versatile and sustainable materials for aquatic environment remediation: A review**

Xiaolin Zhang^a^, Mamitiana Roger Razanajatovo^a^, Xuedong Du^a*^, Shuo Wang^a^, Li Feng^a^, Shunli Wan^c^, Ningyi Chen^d^, Qingrui Zhang^a, b ^[[1]](#footnote-0)^*^

*^a^ Hebei Key Laboratory of Heavy Metal Deep-Remediation in Water and Resource Reuse and Hebei Key Laboratory of Applied Chemistry, School of Environmental and Chemical Engineering, Yanshan University, Qinhuangdao 066004, China.*

*^b^ State Key Laboratory of Metastable Materials Science and Technology, Yanshan University, Qinhuangdao 066004, China.*

*^c^ College of Life & Environment Sciences, Huangshan University, Huangshan 245041, China*

*^d^ College of Environment, Zhejiang University of Technology, Hangzhou 310014, China*

**Table S1.** Basic characteristics of typical proteins for forming amyloid fibrils (ANFs).

| Protein sources | The number of amino acids | Molecular mass (kDa) | The isoelectric point | Remarks | Ref. |
| --- | --- | --- | --- | --- | --- |
| Whey β-lactoglobulin (WβL) | 162 | 18.4 | 5.1-5.3 | The protein contains two disulfide bonds and one free sulfhydryl group, carrying 20 basic amino acids per monomer. | [1] |
| Hen egg-white lysozyme (HEWL) | 129 | 14.3 | 9.5-11 | 17 positively charged (6 Lys, 11 Arg) and nine negatively charged residues (7 Asp, 2 Glu). | [2] |
| Bovine serum albumin (BSA) | 582 | 69 | 4.7 | Ampholyte protein (containing both acidic and basic residues). | [3] |

**Table S2.** Summary of preparation methods and characteristics on amyloid nanofibrils (ANFs) composites.

| Types of composites | Components | Synthesis methods | Morphologies | Functional groups | Ref. |
| --- | --- | --- | --- | --- | --- |
| Combined with polymers | Lysozyme, PDA | The ANFs were prepared by purification and heating. Through post-modification, PDA-induced and cross-linked ANFs. | *D* of fibrils, 80 nm; *S*_BET_, 220.4 m^2^/g | -C=O, -NH_2_ | [4] |
|  | Lysozyme, PDA, PEI | PDA acted as a binder, combining ANFs and PEI together. The reaction temperature was 50 °C. | *D* of fibrils, 40-60 nm; Pore size, 20-500 nm | -OH, -NH_2_, -C=O, -N-H | [5] |
|  | Lysozyme, PEI, g-C_3_N_4_ | The g-C_3_N_4_ was synthesized by calcination, with melamine as the precursor. PEI and g-C_3_N_4_ were mixed by ultrasound. ANFs and the above mixture were assembled during denaturation. | *S*_BET_, 39.6 m^2^/g; Pore size, 13.2 nm | -NH_2_, -COOH, -C=O, -OH, -O-C=NHR | [6] |
|  | β-lactoglobulin, PNiPAM | The composite was prepared by the "bottom-up" method. The anthraquinone-2-sulfonic acid, sodium salt, NiPAM, and BIS were dropped into the ANFs solution. | Hydrodynamic radii, 640 nm; thickness, 22 nm | -C=O, -NH_2_, -O=C-N, -OH | [7] |
|  | Lysozyme, cellulose | The cellulose nanofibrils were prepared by combining mechanical and enzymatic treatments of softwood bisulfite fibers. The suspensions of ANFs and cellulose were directly blended. | A fair dispersion of the lysozyme within the cellulose | -OH, -N-H, -O=C-O, -O=C–N | [8] |
|  | Amyloid fibrils, PDA, cellulose | PDA coated the cellulose. Equal volumes of ANFs and cellulose/PDA were mixed. Sodium periodate promotes the formation of the gel. | Porous aerogel, porosity, 91.1% | -C=O, -C-N, -N-H, -COOH | [9] |
| Combined with metal | Lysozyme, Fe_3_O_4_ | During the fibering process, Fe_3_O_4_ and lysozyme self-assembled at 50 °C for 4 h. | Nano-sized fibrillar structures | Fe^2+^, Fe^3+^ | [10] |
|  | β-lactoglobulin  Fe^0^ | Fe^3+^ was loaded on β-lactoglobulin fibrils through adsorption. And then, Fe^3+^ was reduced to Fe^0^ by NaBH_4_. | *D* of Fe^0^, 5-20 nm | -OH, -NH_2_, -SH, Fe^0^, Fe^2+^, Fe^3+^ | [11] |
|  | β-lactoglobulin, ZrO_2_ | The -NH_2_ of ANFs interacted with Zr^4+^ in solution by strong complexation. Afterward, alkali precipitation promoted the formation of ZrO_2_. | *D* of fibrils, 13-18 nm; *D* of ZrO_2_, 7 nm | -NH_2_, -COOH, Zr^4+^ | [12] |
|  | β-lactoglobulin, Au, Pd | HAuCl_4_ and Na_2_PdCl_4_ were precursors to load Au^3+^ and Pd^2+^ on ANFs. And then, NaBH_4_ played the reduction role. | Length of ANFs, several micrometers | -NH_2_, -COOH, Au^0^, Pd^0^ | [13] |
|  | BSA, Au^+^ | HAuCl_4_ was added during the alkaline denaturation of BSA. | Ellipsoidal particles | -C=O, -N-H, -SH Au^+^ | [14] |
| Combined with carbon | β-lactoglobulin, AC | Mixing the solution of β-lactoglobulin and AC, then vacuum-filtering on the cellulose filter (0.22 µm). | ANFs of several micrometers | -O=C–N, -C=O, -NH_2_, -O=C-O, -OH | [15] |
|  | Whey protein, AC | A solution of Whey fibers was dropped into the solution of AC. The film was prepared by vacuum filtration. | *D* of fibrils, 13 nm | -C=O, -N-H, -NH_2_, -OH | [16] |
|  | Whey protein, carbon | The mixture of whey amyloid and carbohydrate source was put in MQ-water. Afterward, the hydrothermal synthesis was at 50 °C for 5 h. | Aerogel, carbon network; *D* of fibrils, 60 nm; *S*_BET_, 48 m^2^/g | -NH_2_ | [17] |
|  | β-lactoglobulin, AC, paper pulp | The mixture of β-lactoglobulin, AC, and paper pulp was the ratio of 1: 4: 5. The cellulose was used as the support. | Membrane pore size, 0.7 µm | -O=C–N, -C=O, -NH_2_, -O=C-O, -OH | [18] |

PDA, polydopamine; PEI, polyethyleneimine; PNiPAM, poly(N-isopropyl acrylamide); BIS, N, N’-Methylenebis(acrylamide); BSA, bovine serum albumin; AC, activated carbon; *D*, diameter; *S*_BET_, BET specific surface area.

**Table S3.** Comparison of ANFs-based membrane filtration with other methods of bacteria removal.

| Methods of bacteria removal | Cost | Energy consumption | Pressure | Removal mechanisms | Limitations | Ref. |
| --- | --- | --- | --- | --- | --- | --- |
| AHMs | Medium | Medium | - | Size exclusion, hydrogen bonding, hydrophobic, van der Waals and electrostatic interactions | - | [19] |
| Ultrafiltration | Expensive | High | Intensive | Size exclusion techniques | 1. Disrupt the  cell membrane of pathogenic bacteria  2. Lead to the  release of genetic material into water. | [20-22] |
| Nanofiltration |  |  |  |  |  |  |
| Reverse osmosis |  |  |  |  |  |  |
| UV  (ultra-violet) radiation | Cheap | Low | - | Inactivate the  pathogens | Can’t remove genetic material | [23] |
| Chlorination |  |  |  |  |  |  |
| Boiling |  |  |  |  |  |  |
| Ozonation |  |  |  |  |  |  |

AHMs, self-standing amyloid hybrid membranes.

**Fig. S1.** Comparison of typical nanofiber diameters [24-32].


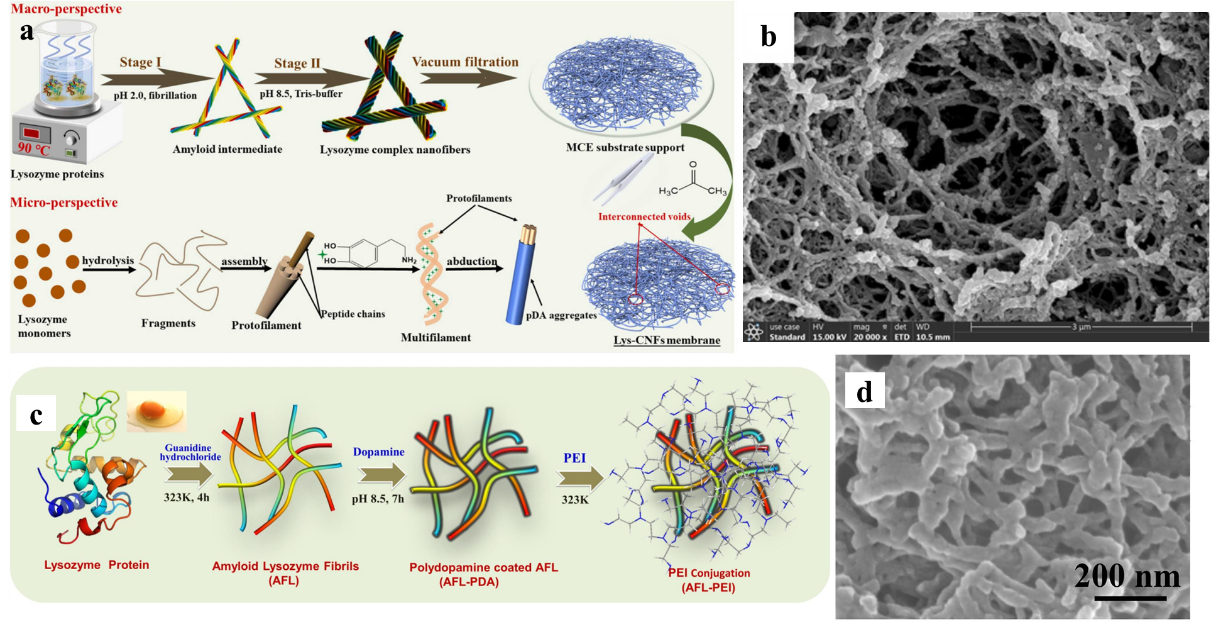


**Fig. S2.** (a) Schematic illustration for preparing Lys-CNFs membrane, (b) SEM image of the magnified details onto Lys-CNFs membrane. Reproduced with permission from Ref. [4]. Copyright (2022) Elsevier. (c) Schematic representation of the synthesis of AFL-PEI nanofibrils, (d) SEM image of the hybrid AFL-PEI. Reproduced with permission from Ref. [5]. Copyright (2020) Elsevier.


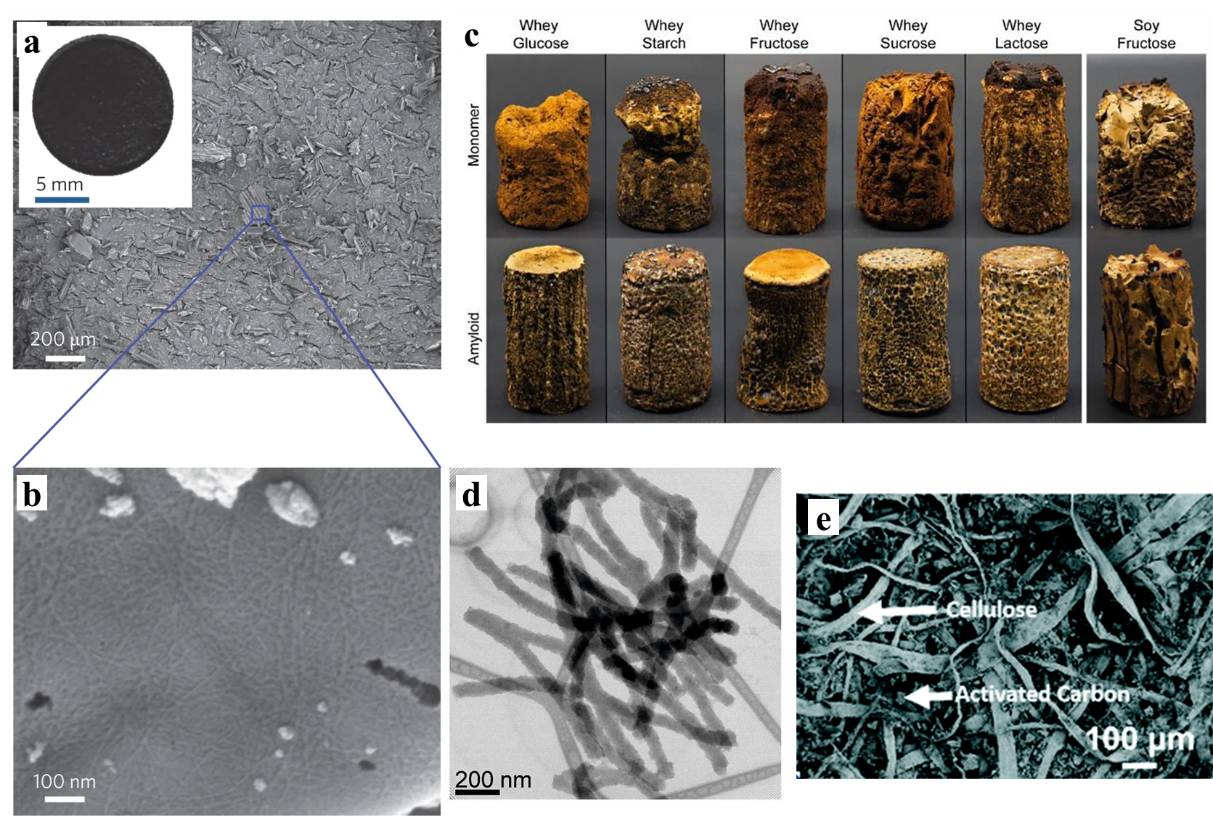


**Fig. S3.** (a) SEM image showing the composite membrane surface, with the visual aspect of the membrane shown in the inset. (b) Higher-magnification SEM image of the membrane, demonstrating the assembly of the amyloid fibrils onto the activated carbon surface. Reproduced with permission from Ref. [15]. Copyright (2017) Springer Nature. (c) Carbon aerogels are produced from different carbohydrates and proteins, such as whey and soy. (d) TEM image of amyloid whey lactose carbon aerogel with 28.5 k magnification. Reproduced with permission from Ref. [17]. Copyright (2022) Elsevier. (e) SEM image of the membrane surface. Reproduced with permission from Ref. [33]. Copyright (2020) The Royal Society of Chemistry.


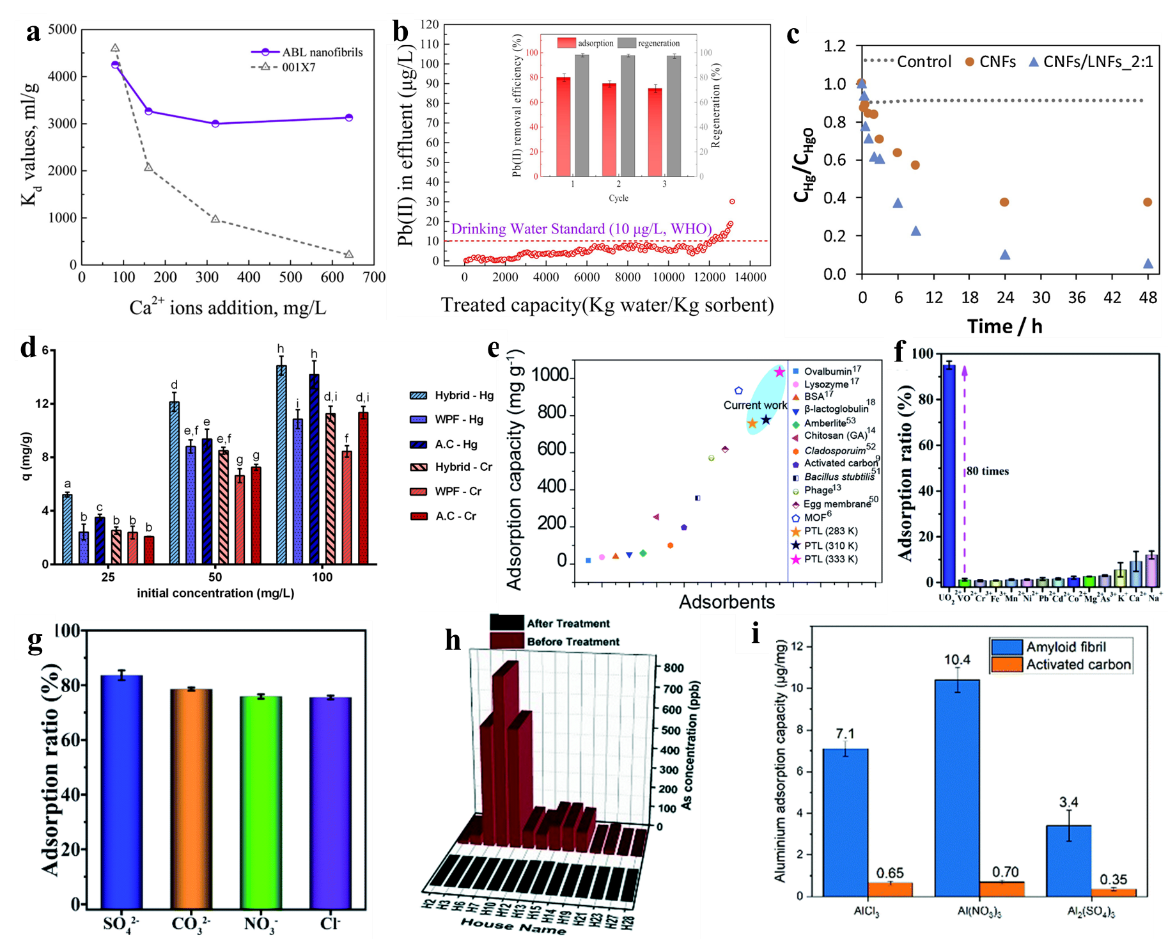


**Fig. S4.** (a) Ion distribution coefficient K_d_ comparison of ABL nanofibrils and 001 × 7. Reproduced with permission from Ref. [34]. Copyright (2020) Elsevier. (b) Continuous treatment capacity evaluation of the AFL-PEI nanofibrils for applications (the embedded figure shows the sorption-regeneration cycles). Reproduced with permission from Ref. [5]. Copyright (2020) Elsevier. (c) Variation of normalized Hg(II), C_Hg_/C_Hg,0_, with time (t, h) for the CNFs and CNFs/LNFs-based films in natural spring water contaminated with 50 μg/L of Hg(II) at pH 11. Reproduced with permission from Ref. [8]. Copyright (2020) Elsevier. (d) Adsorption capacity of membranes of AC, WPF, and the hybrid of 1:2 AC: WPF for mercury (blue bars) and chromium (red bars) at initial concentrations of 25, 50, and 100 mg/L. Reproduced with permission from Ref. [16]. Copyright (2020) MDPI. (e) Comparison of gold adsorption capacity among different adsorbents. Reproduced with permission from Ref. [35]. Copyright (2020) The Royal Society of Chemistry. (f) The effect of competing metal ions (1 ppm each) on uranium ion adsorption. (g) The effect of Cl^-^, CO_3_^2-^, SO_4_^2-^ and NO_3_^-^ (0.01 M) on uranium ion adsorption. Reproduced with permission from Ref. [36]. Copyright (2022) The Royal Society of Chemistry. (h) As concentration before and after filtration with the type I household filtration unit (Tacna: H2, H3, H10, Ticapampa: H6, H7, Inclan: H12, H13, H14, H15, Chiclayo: H19, Moquegua: H21, and Cerro de Pasco: H27, H28). Reproduced with permission from Ref. [37]. Copyright (2021) The Royal Society of Chemistry. (i) Comparison of the relative specific adsorption capacities per mg of amyloid fibrils and/or activated carbon membranes. Reproduced with permission from Ref. [38]. Copyright (2019) The Royal Society of Chemistry.


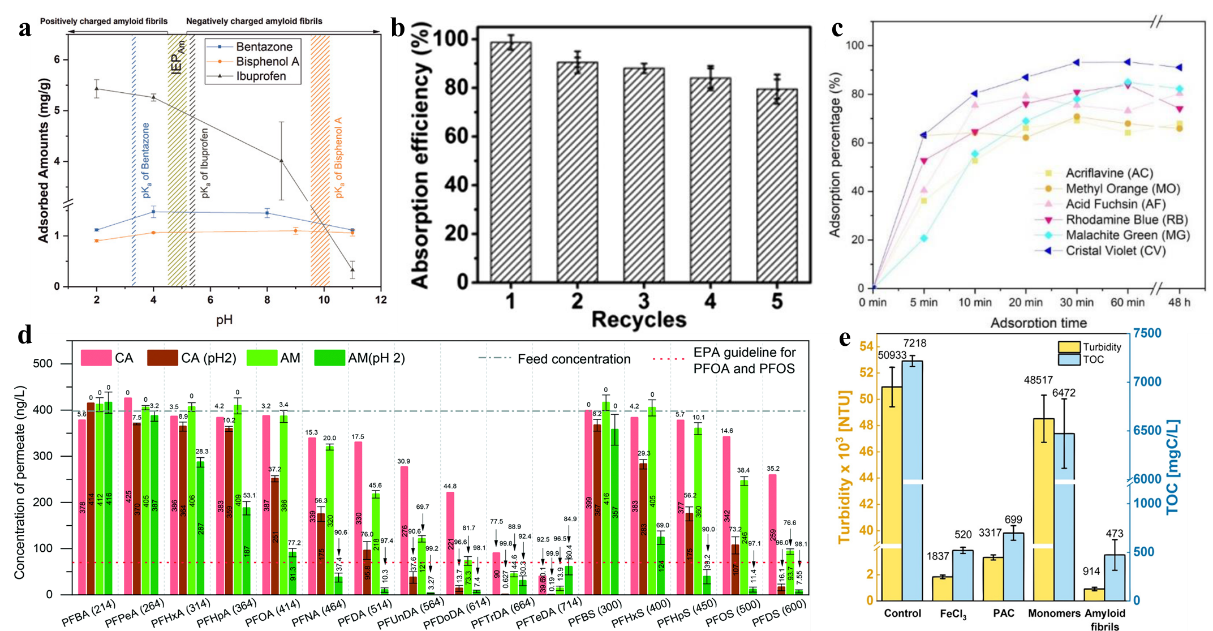


**Fig. S5.** (a) Effect of pH on organic pollutants adsorption by amyloid fibrils aerogel. Reproduced with permission from Ref. [39]. Copyright (2020) Wiley. (b) Trapping stability of biofilms to congo red. Reproduced with permission from Ref. [40]. Copyright (2018) Elsevier. (c) Dye adsorption percentage of CpA aerogel as a function of time. Reproduced with permission from Ref. [9]. Copyright (2021) MDPI. (d) Concentrations of PFCAs and PFSAs in the permeate. The pH of the feed is around 7 unless otherwise stated. The number in the brackets is the MW of each compound. Reproduced with permission from Ref. [41]. Copyright (2021) The Royal Society of Chemistry. (e) Comparison of commercial flocculants, FeCl_3_ and PAC, lysozyme protein monomers, and lysozyme amyloid fibrils for removing dispersed polystyrene MPs from water by measuring turbidity and TOC. Reproduced with permission from Ref. [24]. Copyright (2021) American Chemical Society.

**References**

[1] J.-M. Jung, G. Savin, M. Pouzot, C. Schmitt, R. Mezzenga, Structure of heat-induced β-lactoglobulin aggregates and their complexes with sodium-dodecyl sulfate, Biomacromolecules 9(9) (2008) 2477-2486. <https://doi.org/10.1021/bm800502j>.

[2] T. Wu, Q. Jiang, D. Wu, Y. Hu, S. Chen, T. Ding, X. Ye, D. Liu, J. Chen, What is new in lysozyme research and its application in food industry? A review, Food Chem. 274 (2019) 698-709. <https://doi.org/10.1016/j.foodchem.2018.09.017>.

[3] K. Jaglińska, B. Polak, A. Klimek-Turek, P. Pomastowski, B. Buszewski, T.H. Dzido, Retardation of some drugs in thin-layer chromatographic systems with impregnated silica gel plates with hen's egg white and bovine serum albumin, J. Chromatogr. A. 1625 (2020) 461277. <https://doi.org/10.1016/j.chroma.2020.461277>.

[4] C. Liang, L. Zhao, L. Qiao, K. Du, Proteinaceous porous nanofiber membrane-type adsorbent derived from amyloid lysozyme protofilaments for highly efficient lead(II) biologic scavenging, J. Hazard. Mater. 425 (2022) 127886. <https://doi.org/10.1016/j.jhazmat.2021.127886>.

[5] M. Liu, L. Jia, Z. Zhao, Y. Han, Y. Li, Q. Peng, Q. Zhang, Fast and robust lead (II) removal from water by bioinspired amyloid lysozyme fibrils conjugated with polyethyleneimine (PEI), Chem. Eng. J. 390 (2020) 124667. <https://doi.org/10.1016/j.cej.2020.124667>.

[6] E. Arputharaj, A.S. Krishna Kumar, W.-L. Tseng, S.-J. Jiang, Y.-L. Huang, H.-U. Dahms, Self-assembly of poly(ethyleneimine)-modified g-C_3_N_4_ nanosheets with lysozyme fibrils for chromium detoxification, Langmuir 37(23) (2021) 7147-7155. <https://doi.org/10.1021/acs.langmuir.1c00716>.

[7] C. Li, M.M. Alam, S. Bolisetty, J. Adamcik, R. Mezzenga, New biocompatible thermo-reversible hydrogels from PNiPAM-decorated amyloid fibrils, Chem. Commun. 47(10) (2011) 2913-2915. <https://doi.org/10.1039/C0CC05126H>.

[8] N.H.C.S. Silva, P. Figueira, E. Fabre, R.J.B. Pinto, M.E. Pereira, A.J.D. Silvestre, I.M. Marrucho, C. Vilela, C.S.R. Freire, Dual nanofibrillar-based bio-sorbent films composed of nanocellulose and lysozyme nanofibrils for mercury removal from spring waters, Carbohyd. Polym. 238 (2020) 116210. <https://doi.org/10.1016/j.carbpol.2020.116210>.

[9] M. Sorriaux, M. Sorieul, Y. Chen, Bio-based and robust polydopamine coated nanocellulose/amyloid composite aerogel for fast and wide-spectrum water purification, Polymers 13(19) (2021) 3442. <https://doi.org/10.3390/polym13193442>.

[10] W.-H. Leung, W.-H. Lo, P.-H. Chan, Amyloid fibrils as rapid and efficient nano-biosorbents for removal of dye pollutants, RSC Adv. 5(109) (2015) 90022-90030. <https://doi.org/10.1039/C5RA17182B>.

[11] Y. Shen, L. Posavec, S. Bolisetty, F.M. Hilty, G. Nyström, J. Kohlbrecher, M. Hilbe, A. Rossi, J. Baumgartner, M.B. Zimmermann, R. Mezzenga, Amyloid fibril systems reduce, stabilize and deliver bioavailable nanosized iron, Nat. Nanotech. 12(7) (2017) 642-647. <https://doi.org/10.1038/nnano.2017.58>.

[12] Q. Zhang, S. Bolisetty, Y. Cao, S. Handschin, J. Adamcik, Q. Peng, R. Mezzenga, Selective and efficient removal of fluoride from water: In situ engineered amyloid fibril/ZrO_2_ hybrid membranes, Angew. Chem. Int. Ed. 58(18) (2019) 6012-6016. <https://doi.org/10.1002/anie.201901596>.

[13] S. Bolisetty, M. Arcari, J. Adamcik, R. Mezzenga, Hybrid amyloid membranes for continuous flow catalysis, Langmuir 31(51) (2015) 13867-13873. <https://doi.org/10.1021/acs.langmuir.5b03205>.

[14] X. Yu, W. Liu, X. Deng, S. Yan, Z. Su, Gold nanocluster embedded bovine serum albumin nanofibers-graphene hybrid membranes for the efficient detection and separation of mercury ion, Chem. Eng. J. 335 (2018) 176-184. <https://doi.org/10.1016/j.cej.2017.10.148>.

[15] S. Bolisetty, R. Mezzenga, Amyloid–carbon hybrid membranes for universal water purification, Nat. Nanotech. 11(4) (2016) 365-371. <https://doi.org/10.1038/nnano.2015.310>.

[16] L.C. Ramírez-Rodríguez, L.E. Díaz Barrera, M.X. Quintanilla-Carvajal, D.I. Mendoza-Castillo, A. Bonilla-Petriciolet, C. Jiménez-Junca, Preparation of a hybrid membrane from whey protein fibrils and activated carbon to remove mercury and chromium from water, Membranes 10(12) (2020) 386. <https://doi.org/10.3390/membranes10120386>.

[17] M. Peydayesh, J. Vogt, X. Chen, J. Zhou, F. Donat, M. Bagnani, C.R. Müller, R. Mezzenga, Amyloid-based carbon aerogels for water purification, Chem. Eng. J. 449 (2022) 137703. <https://doi.org/10.1016/j.cej.2022.137703>.

[18] N.M. Chiera, S. Bolisetty, R. Eichler, R. Mezzenga, P. Steinegger, Removal of radioactive cesium from contaminated water by whey protein amyloids–carbon hybrid filters, RSC Adv. 11(51) (2021) 32454-32458. <https://doi.org/10.1039/D1RA05376K>.

[19] A. Palika, A. Rahimi, S. Bolisetty, S. Handschin, P. Fischer, R. Mezzenga, Amyloid hybrid membranes for bacterial & genetic material removal from water and their anti-biofouling properties, Nanoscale Adv. 2(10) (2020) 4665-4670. <https://doi.org/10.1039/D0NA00189A>.

[20] J. Zheng, C. Su, J. Zhou, L. Xu, Y. Qian, H. Chen, Effects and mechanisms of ultraviolet, chlorination, and ozone disinfection on antibiotic resistance genes in secondary effluents of municipal wastewater treatment plants, Chem. Eng. J. 317 (2017) 309-316. <https://doi.org/10.1016/j.cej.2017.02.076>.

[21] W.A.M. Hijnen, E.F. Beerendonk, G.J. Medema, Inactivation credit of UV radiation for viruses, bacteria and protozoan (oo)cysts in water: A review, Water Res. 40(1) (2006) 3-22. <https://doi.org/https://doi.org/10.1016/j.watres.2005.10.030>.

[22] X. Li, M. Cai, L. Wang, F. Niu, D. Yang, G. Zhang, Evaluation survey of microbial disinfection methods in UV-LED water treatment systems, Sci. Total Environ. 659 (2019) 1415-1427. <https://doi.org/https://doi.org/10.1016/j.scitotenv.2018.12.344>.

[23] H.-C. Su, Y.-S. Liu, C.-G. Pan, J. Chen, L.-Y. He, G.-G. Ying, Persistence of antibiotic resistance genes and bacterial community changes in drinking water treatment system: From drinking water source to tap water, Sci. Total Environ. 616-617 (2018) 453-461. <https://doi.org/https://doi.org/10.1016/j.scitotenv.2017.10.318>.

[24] M. Peydayesh, T. Suta, M. Usuelli, S. Handschin, G. Canelli, M. Bagnani, R. Mezzenga, Sustainable removal of microplastics and natural organic matter from water by coagulation–flocculation with protein amyloid fibrils, Environ. Sci. Technol. 55(13) (2021) 8848-8858. <https://doi.org/10.1021/acs.est.1c01918>.

[25] X. Hu, C. Zou, X. Zou, The formation of supramolecular carbon nanofiber via amidation reaction on the surface of amino single walled carbon nanotubes for selective adsorption organic pollutants, J. Colloid Interface Sci. 542 (2019) 112-122. <https://doi.org/10.1016/j.jcis.2019.01.130>.

[26] G. Chizari Fard, M. Mirjalili, F. Najafi, Hydroxylated α-Fe_2_O_3_ nanofiber: Optimization of synthesis conditions, anionic dyes adsorption kinetic, isotherm and error analysis, J. Taiwan Inst. Chem. Eng. 70 (2017) 188-199. <https://doi.org/10.1016/j.jtice.2016.10.045>.

[27] L. Li, Y. Li, L. Cao, C. Yang, Enhanced chromium (VI) adsorption using nanosized chitosan fibers tailored by electrospinning, Carbohyd. Polym. 125 (2015) 206-213. <https://doi.org/10.1016/j.carbpol.2015.02.037>.

[28] H. Sun, J. Feng, Y. Song, L. Xu, X. Cui, B. Yu, Preparation of the carbonized Zif-8@PAN nanofiber membrane for cadmium ion adsorption, Polymers 14(13) (2022) 2523. <https://doi.org/10.3390/polym14132523>.

[29] A. Mahapatra, B.G. Mishra, G. Hota, Electrospun Fe_2_O_3_–Al_2_O_3_ nanocomposite fibers as efficient adsorbent for removal of heavy metal ions from aqueous solution, J. Hazard. Mater. 258-259 (2013) 116-123. <https://doi.org/10.1016/j.jhazmat.2013.04.045>.

[30] Y. Lu, W. Zhang, M. Wang, H. Zhang, J. Li, W. Luo, Fabrication of GO/PAN nanofiber membrane grafted with chitosan as efficient adsorbent for dye removal, J. Polym. Environ. 30(7) (2022) 2943-2954. <https://doi.org/10.1007/s10924-022-02393-6>.

[31] W. Guo, R. Guo, H. Pei, B. Wang, N. Liu, Z. Mo, PAN/PEI nanofiber membrane for effective removal of heavy metal ions and oil–water separation, J. Polym. Environ. 30(11) (2022) 4835-4847. <https://doi.org/10.1007/s10924-022-02541-y>.

[32] H.Y. Choi, J.H. Bae, Y. Hasegawa, S. An, I.S. Kim, H. Lee, M. Kim, Thiol-functionalized cellulose nanofiber membranes for the effective adsorption of heavy metal ions in water, Carbohyd. Polym. 234 (2020) 115881. <https://doi.org/10.1016/j.carbpol.2020.115881>.

[33] S. Bolisetty, N.M. Coray, A. Palika, G.A. Prenosil, R. Mezzenga, Amyloid hybrid membranes for removal of clinical and nuclear radioactive wastewater, Environ. Sci.: Water Res. Technol. 6(12) (2020) 3249-3254. <https://doi.org/10.1039/D0EW00693A>.

[34] Q. Zhang, S. Zhang, Z. Zhao, M. Liu, X. Yin, Y. Zhou, Y. Wu, Q. Peng, Highly effective lead (II) removal by sustainable alkaline activated β-lactoglobulin nanofibrils from whey protein, J. Clean. Prod. 255 (2020) 120297. <https://doi.org/10.1016/j.jclepro.2020.120297>.

[35] F. Yang, Z. Yan, J. Zhao, S. Miao, D. Wang, P. Yang, Rapid capture of trace precious metals by amyloid-like protein membrane with high adsorption capacity and selectivity, J. Mater. Chem. A. 8(6) (2020) 3438-3449. <https://doi.org/10.1039/C9TA12124B>.

[36] Q. Yang, J. Zhao, A. Muhammad, R. Qin, J. Tian, L. Li, Q. Zhang, L. Chen, P. Yang, An amyloid-like proteinaceous adsorbent for uranium extraction from aqueous medium, J. Mater. Chem. A. 10(28) (2022) 14906-14916. <https://doi.org/10.1039/D2TA02342C>.

[37] S. Bolisetty, A. Rahimi, R. Mezzenga, Arsenic removal from Peruvian drinking water using milk protein nanofibril–carbon filters: a field study, Environ. Sci.: Water Res. Technol. 7(12) (2021) 2223-2230. <https://doi.org/10.1039/D1EW00456E>.

[38] M. Peydayesh, M. Pauchard, S. Bolisetty, F. Stellacci, R. Mezzenga, Ubiquitous aluminium contamination in water and amyloid hybrid membranes as a sustainable possible solution, Chem. Commun. 55(74) (2019) 11143-11146. <https://doi.org/10.1039/C9CC05337A>.

[39] M. Peydayesh, M.K. Suter, S. Bolisetty, S. Boulos, S. Handschin, L. Nyström, R. Mezzenga, Amyloid fibrils aerogel for sustainable removal of organic contaminants from water, Adv. Mater. 32(12) (2020) 1907932. <https://doi.org/10.1002/adma.201907932>.

[40] X. Wu, X. Han, L. Lv, M. Li, J. You, C. Li, Supramolecular proteinaceous biofilms as trapping sponges for biologic water treatment and durable catalysis, J. Colloid Interface Sci. 527 (2018) 117-123. <https://doi.org/10.1016/j.jcis.2018.05.036>.

[41] T. Jin, M. Peydayesh, H. Joerss, J. Zhou, S. Bolisetty, R. Mezzenga, Amyloid fibril-based membranes for PFAS removal from water, Environ. Sci.: Water Res. Technol. 7(10) (2021) 1873-1884. <https://doi.org/10.1039/D1EW00373A>.

1. * Corresponding authors: duxd@ysu.edu.cn (Xuedong Du), zhangqr@ysu.edu.cn (Qingrui Zhang) [↑](#footnote-ref-0)
